# Multivalent poultry vaccine development using Protein Glycan Coupling Technology

**DOI:** 10.1186/s12934-021-01682-4

**Published:** 2021-10-02

**Authors:** Marta Mauri, Thippeswamy H. Sannasiddappa, Prerna Vohra, Ricardo Corona-Torres, Alexander A. Smith, Cosmin Chintoan-Uta, Abi Bremner, Vanessa S. Terra, Sherif Abouelhadid, Mark P. Stevens, Andrew J. Grant, Jon Cuccui, Brendan W. Wren, the Glycoengineering of Veterinary Vaccines Consortium

**Affiliations:** 1 Department of Infection Biology, London School of Hygiene and Tropical Medicine, Keppel Street, London, WC1E 7HT UK; 2 Department of Veterinary Medicine, University of Cambridge, Madingley Road, Cambridge, CB3 0ES Cambridgeshire UK; 3 The Roslin Institute, University of Edinburgh, Easter Bush Campus, Midlothian, Edinburgh, EH25 9RG UK; 4 Institute for Immunology and Infection Research, School of Biological Sciences, University of Edinburgh, Charlotte Auerbach Road, Edinburgh, EH9 3FL UK

**Keywords:** Vaccine, Glycoconjugates, PGCT, Glycoengineering, Live-attenuated, Poultry, One Health, *Campylobacter jejuni*, APEC, NetB

## Abstract

**Background:**

Poultry is the world's most popular animal-based food and global production has tripled in the past 20 years alone. Low-cost vaccines that can be combined to protect poultry against multiple infections are a current global imperative. Glycoconjugate vaccines, which consist of an immunogenic protein covalently coupled to glycan antigens of the targeted pathogen, have a proven track record in human vaccinology, but have yet to be used for livestock due to prohibitively high manufacturing costs. To overcome this, we use Protein Glycan Coupling Technology (PGCT), which enables the production of glycoconjugates in bacterial cells at considerably reduced costs, to generate a candidate glycan-based live vaccine intended to simultaneously protect against *Campylobacter jejuni*, avian pathogenic *Escherichia coli* (APEC) and *Clostridium perfringens*. *Campylobacter* is the most common cause of food poisoning, whereas colibacillosis and necrotic enteritis are widespread and devastating infectious diseases in poultry.

**Results:**

We demonstrate the functional transfer of *C. jejuni* protein glycosylation (*pgl*) locus into the genome of APEC χ7122 serotype O78:H9. The integration caused mild attenuation of the χ7122 strain following oral inoculation of chickens without impairing its ability to colonise the respiratory tract. We exploit the χ7122 *pgl* integrant as bacterial vectors delivering a glycoprotein decorated with the *C. jejuni* heptasaccharide glycan antigen. To this end we engineered χ7122 *pgl* to express glycosylated NetB toxoid from *C. perfringens* and tested its ability to reduce caecal colonisation of chickens by *C. jejuni* and protect against intra-air sac challenge with the homologous APEC strain.

**Conclusions:**

We generated a candidate glycan-based multivalent live vaccine with the potential to induce protection against key avian and zoonotic pathogens (*C. jejuni*, APEC, *C. perfringens*). The live vaccine failed to significantly reduce *Campylobacter* colonisation under the conditions tested but was protective against homologous APEC challenge. Nevertheless, we present a strategy towards the production of low-cost “live-attenuated multivalent vaccine factories” with the ability to express glycoconjugates in poultry.

**Supplementary Information:**

The online version contains supplementary material available at 10.1186/s12934-021-01682-4.

## Background

Healthily maintained livestock are essential for economic and societal prosperity [1]. Poultry are the main source of meat and eggs worldwide. The world's chicken flock is now estimated to be around 66 billion broilers and 21 billion layers [2]. Poultry meat production has grown 12-fold in the past 50 years [2]. Additionally, poultry accounts for egg production, which globally has increased threefold in the last three decades with c. 87 billion eggs estimated to be produced per annum [3]. However, infectious diseases remain a significant impediment to poultry welfare and productivity. Consequently, there is a growing demand to identify successful strategies to prevent the spread of diseases within and from flocks, to avert significant economic losses and to mitigate the healthcare burden resulting from zoonoses arising from poultry products.

The most common bacterial infections encountered in poultry are colibacillosis, mycoplasmosis, and salmonellosis, caused by avian pathogenic *E. coli* (APEC), *Mycoplasma gallisepticum* (and less frequently by *Mycoplasma synoviae* and *Mycoplasma meleagridis*), and *Salmonella enterica* species (mostly *Salmonella enterica* serovar Pullorum and *Salmonella enterica* serovar Gallinarum), respectively [4]. Other less common, but possibly severe bacterial infections include fowl cholera, necrotic enteritis, botulism and tuberculosis [4]. Aside from contracting infections, poultry can also transmit zoonotic diseases of public health concern to humans, such as campylobacteriosis, salmonellosis, and avian influenza viruses causing gastroenteritis, diarrhoea and fever [5]. In Europe and the UK, for instance, bacterial species of *Campylobacter* and *Salmonella* are the top two reported bacterial gastrointestinal pathogens in humans. These are WHO-listed high priority pathogens given the rise of antibiotic resistant species, and undercooked chicken meat and eggs are key sources of human infection [6–8]. The diseases they cause are usually self-limiting in people, but in severe cases they require hospitalisation, and can result in death, generally posing a higher threat for children younger than 5 years, people with weakened immune systems, pregnant women and the elderly [5]. Worse outcomes, particularly in young children, are more common in poor settings lacking safe water, effective sanitation, standard hygiene, and hospital access, emphasising the global health concerns around zoonotic enteritis and the importance of the One Health approach (health for people, animals and the environment) to tackle them [6, 9, 10].

General recommendations to reduce the spread of infectious diseases are the implementation of generic hygiene measures, together with safe cooking and food handling practices to avoid consumption of raw or undercooked animal products [11]. Alongside these practices, the introduction of vaccines has been one of the most impactful and cost-effective public health measures to prevent the spread of diseases (reviewed in [12]), saving an estimated 2–3 million lives each year [13]. Amongst the different types of vaccines that exist to date, glycoconjugates, consisting of a glycan antigen from the surface of the target pathogen covalently coupled to a protein carrier with strong immunogenic properties, have a proven track record in human vaccinology for safety and efficacy [14]. In fact, since the introduction of licensed glycoconjugate vaccines against *Haemophilus influenzae* type b, *Streptococcus pneumoniae,* and *Neisseria meningitidis* from the 1980s, the incidence of pneumonia and meningitis has dramatically decreased [12, 15]. The key element of their effectiveness in inducing durable B- and T-cell responses is the coupling of the glycan and protein moieties, a crucial observation that stemmed from pioneering work of Avery and Goebbels in the 1920s [16]. Linking a carrier protein to a glycan antigen overcomes the limitations of polysaccharide-only vaccines, which fail to induce protection in infants younger than 18 months, are T-cell independent antigens and fail to induce immunological memory [17–19]. The traditional production of glycoconjugates, however, is a convoluted multi-step process that requires large scale cultures of pathogenic microorganisms as a source of the glycan antigen or chemical synthesis of the glycan, the separate production of a recombinant carrier protein, and the chemical or enzymatic coupling of the two. The complexity of the production process is reflected in high costs that has so far hindered their use in the veterinary field.

To circumvent cost and biosafety issues, we employ Protein Glycan Coupling Technology (PGCT), which utilises modified bacterial strains to produce glycoconjugates recombinantly in vivo (reviewed in [20, 21]). This biotechnology stemmed from the discovery of an *N*-linked protein glycosylation locus (*pgl*) in the bacterium *C. jejuni* [22, 23] and its functional transfer to *E. coli* [24]. The subsequent observations that the oligosaccharyltransferase (OST) of the *pgl* locus, PglB, has relaxed specificity for its glycan substrate [25] and the identification of a relatively short PglB acceptor sequon, D/EXNXS/T (where X is any amino acid except proline) [26], paved the way for exploiting PGCT for glycoconjugate production. Since then this process, also known as bioconjugation, has been continuously improved and used to produce multiple candidate glycoconjugate vaccines from safe laboratory-adapted strains of *E. coli* (for a comprehensive list see Table 3 in [20]). The recombinant expression of glycoproteins is usually achieved by transforming suitable *E. coli* strains with plasmids encoding the functional components of the system: the glycan antigen, a carrier protein of choice and an OST capable of coupling the two. Alternatively, these components can be chromosomally integrated to overcome the use of plasmids. Interestingly, PGCT has already been successfully employed to produce recombinant vaccine candidates for poultry. Work form the Szymanski group has shown that a diphtheria toxin fused to a *C. jejuni* peptide containing nine acceptor sequons modified with *C. jejuni* heptasaccharide significantly reduced *Campylobacter* colonisation of the chicken gut [27]. A prior study from our group generated SodB and FlpA glycoconjugates where both protein and glycan antigen target *Campylobacter* species [28]. These candidates failed to reduce *Campylobacter* colonisation in the chosen vaccination schedule, possibly owing to lower levels of protein glycosylation than reported by others but are undergoing further improvements to boost their immunogenicity.

PGCT is not limited to glycoengineering strains of *E. coli*, but it can be applied to other Gram-negative bacteria circumventing the need for heterologous glycan locus expression, which can be challenging. Successful examples in this respect include the application of PGCT to *Shigella flexneri 2a* [29], *Salmonella enterica* serovar Paratyphi [30] and *Yersinia enterocolitica* O9, which shares the same glycan structure of *Brucella suis* and *Brucella abortus*, while belonging to a lower biohazard level [31, 32].

In this study we utilise PGCT to develop a multivalent live vaccine for poultry capable of expressing heterologous glycoproteins in vitro and in vivo. The modified APEC live vaccine is exploited as a vector for the delivery of the *Clostridium perfringens* NetB toxoid coupled to the *Campylobacter jejuni* heptasaccharide glycan. Thus, the candidate trivalent vaccine has the potential to protect poultry from colibacillosis and necrotic enteritis caused by APEC and *C. perfringens*, respectively, and to reduce *Campylobacter* colonisation of the chicken gut to prevent zoonotic gastroenteritis. As a bacterial vector we chose a widely studied APEC strain (χ7122) from a serotype (O78:H9) and sequence type (ST23) that is commonly associated with colibacillosis in poultry worldwide [33]. We transferred the *pgl* locus from *C. jejuni*, which encodes a conserved heptasaccharide glycan expressed by all thermophilic *Campylobacter* species (including *C. jejuni* and *C. coli*, which are the most reported causes of human gastroenteritis) [34] to the chromosome of χ7122 and confirmed it to be functional. The *N*-linked heptasaccharide glycan decorates around 50 periplasmic and surface proteins in its natural *C. jejuni* host [35], where abrogation of *N*-linked glycosylation causes proteome instability and impairs the ability of the bacteria to colonise poultry, highlighting an important function of this moiety [36, 37]. Because of its conservation across enteropathogenic *Campylobacters* [34], its key biological function [36–38], surface exposure [39], proven immunogenicity in rabbits [34], recognition by a human lectin [40], and prior success in vaccine formulations [41–43], this glycan was chosen as a vaccine antigen for this study. The heptasaccharide was then coupled by the PglB enzyme to the chosen acceptor protein, a detoxified variant of the *C. perfringens* pore-forming toxin NetB, which was modified to be shuttled to the periplasm (where PglB functions) and to contain ten glycan acceptor sequons. NetB is associated with the pathogenesis of necrotic enteritis and a promising vaccine candidate, as demonstrated by its prior use in a *Salmonella*-vectored vaccine [44].

The χ7122 *pgl* integrant expressing glycosylated NetB described herein was tested in a chicken model to assess in vivo fitness and its ability to reduce *Campylobacter* colonisation of the caeca and to protect from respiratory APEC infection.

## Results

### Generation of a suicide plasmid for the chromosomal integration of *C. jejuni* protein glycosylation (*pgl*) locus in avian pathogenic *E. coli*

**Fig. 1:**
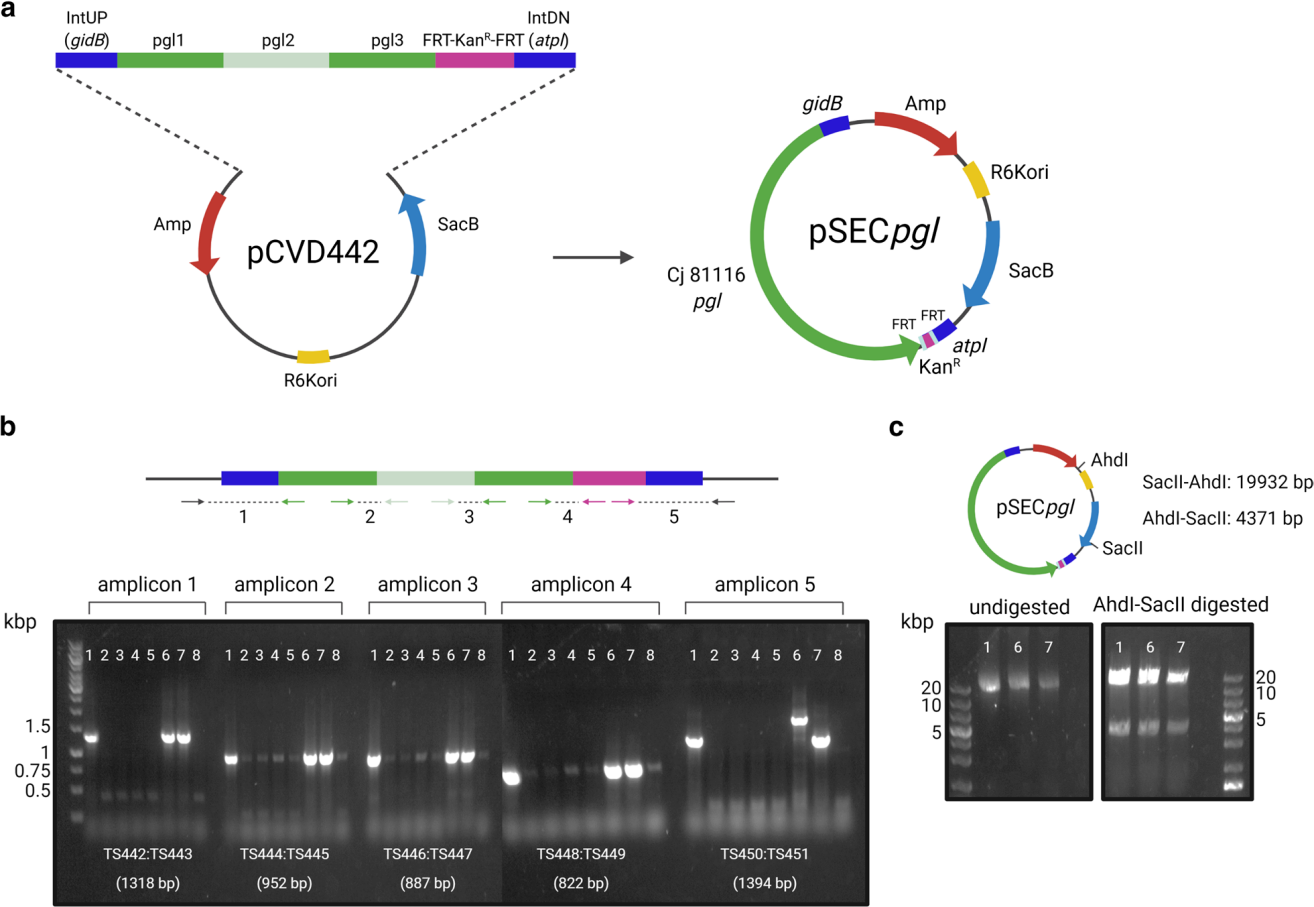
Generation of a suicide plasmid for chromosomal integration of the *C. jejuni pgl* locus in APEC χ7122 via homologous recombination **a** Schematic representation of the cloning strategy used to construct the pSEC*pgl* suicide vector used for integration of the *C. jejuni pgl* locus between genes *gidB* and *atpI* in APEC genome. A Gibson assembly reaction with seven fragments was assembled. IntUP (~ 1 kb left homology arm for integration), *C. jejuni pgl* operon consisting of 3 fragments of ~ 5 kb each (pgl1-3), kanamycin selection marker surrounded by FRT sites, and IntDN (~ 1 kb right homology arm for integration) were PCR amplified and assembled into linearised pCVD442 plasmid between base coordinates 3633 and 4253 replacing the IS1 element. **b** Junction PCRs to assess correct assembly of pSEC*pgl*. Lanes 1 to 8—diagnostic junction PCR products from eight Amp^R^, Kan^R^ Gibson assembly transformants. The expected PCR product size along with primer pairs used are shown. Clones 1 and 7 showed expected PCR products for all five junction PCRs tested. **c)**Diagnostic restriction analysis of pSEC*pgl* plasmids isolated from colonies 1, 6 and 7 shown in **b**

To generate a multivalent live vaccine candidate for poultry using PGCT, we elected to genetically engineer a well-studied strain of APEC to express the *C. jejuni* heptasaccharide as a glycan antigen to reduce *Campylobacter* colonisation of chicken intestines, and consequently decrease poultry-derived food poisoning. In order to avoid placing an excessive metabolic burden on the APEC vector and to avoid having to use selective antibiotics to culture the vaccine strain, we devised a strategy for chromosomal integration of the *C. jejuni pgl* locus in the genome of χ7122, serotype O78:H9 [45]. Previous studies have shown that expression of the *pgl* locus and glycosylation of the targeted proteins is achieved in *E. coli* K-12 strains when both components are expressed from plasmids under the control of the *pgl* endogenous promoters and inducible promoters, respectively [24, 28, 38, 41, 46]. More recently, the *pgl* genes have been integrated in the genome of *E. coli* K-12 MG1655 with demonstrated functionality and improved growth phenotypes, indicating that a single chromosomal copy of the *C. jejuni* glycosylation machinery is sufficient to perform *N*-linked glycosylation of chosen acceptor proteins in the absence of significant metabolic burden on cell growth [47]. To this end, we constructed a suicide vector carrying the *C. jejuni pgl* locus terminally marked with a kanamycin resistance cassette surrounded by flippase recognition target (FRT) sites for flippase-mediated removal of the antibiotic marker. The tagged *pgl* locus was flanked by two ~ 1 kb long homology arms (IntUP and IntDN) for integration into the APEC recipient strain by allelic exchange. The homology arms result in the introduction of the *pgl* cassette into a pseudogene, with the loss of 12 bp of the pseudogene between genes *gidB* and *atpI* (bases 3993380 and 3997791), an integration site that was previously shown to promote efficient expression of a reporter protein [48]. pCVD442 was used as a suicide vector backbone as it carries a *pir*-dependent R6K origin of replication, requiring λ*pir* + strains for its maintenance, and the *sacB* gene of *Bacillus subtilis* conferring sensitivity to sucrose, allowing positive selection for double recombinants that have lost the vector and carry the endogenous copy of the target region [49]. A Gibson assembly reaction [50] with seven fragments spanning the IntUP and IntDN homology arms for integration, the *pgl* locus divided into three 5 kb pieces, the FRT-flanked kanamycin marker, and the linearised pCVD442 vector, was constructed, resulting in plasmid pSEC*pgl* (Fig. 1a). The assembly was electroporated into competent *E. coli* S17-1 λ*pir* cells. Eight transformants growing on selective agar containing ampicillin (pCVD442-encoded resistance marker) and kanamycin (resistance marker tagging the *pgl* locus) were screened by colony PCR with primers amplifying the fragment junctions to assess plasmid assembly. The resulting PCR amplicons were analysed by 1% DNA agarose gel electrophoresis, identifying colonies 1 and 7 as correctly assembled clones given that all five amplicons showed the expected size (Fig. 1b). Purified pSEC*pgl* plasmids from colonies 1, 6, and 7 were also checked by restriction digest with AhdI-SacII restriction enzymes to confirm the integrity of the assembled plasmids. All three clones showed the expected restriction pattern of fragments at 19,932 and 4371 bp (Fig. 1c), however only clones 1 and 7 were confirmed to be correct by paired-end Illumina sequencing. These two plasmids were compared against the expected in silico reference sequence to identify single nucleotide polymorphisms (SNPs) and insertion/deletion (indel) variants. Both plasmids had a single nucleotide base change from T to C, at base coordinate 19,014. This change was outside of the coding and regulatory regions of the plasmid; hence it was deemed acceptable.

### Integration of the *C. jejuni pgl* locus in the APEC genome

**Fig. 2:**
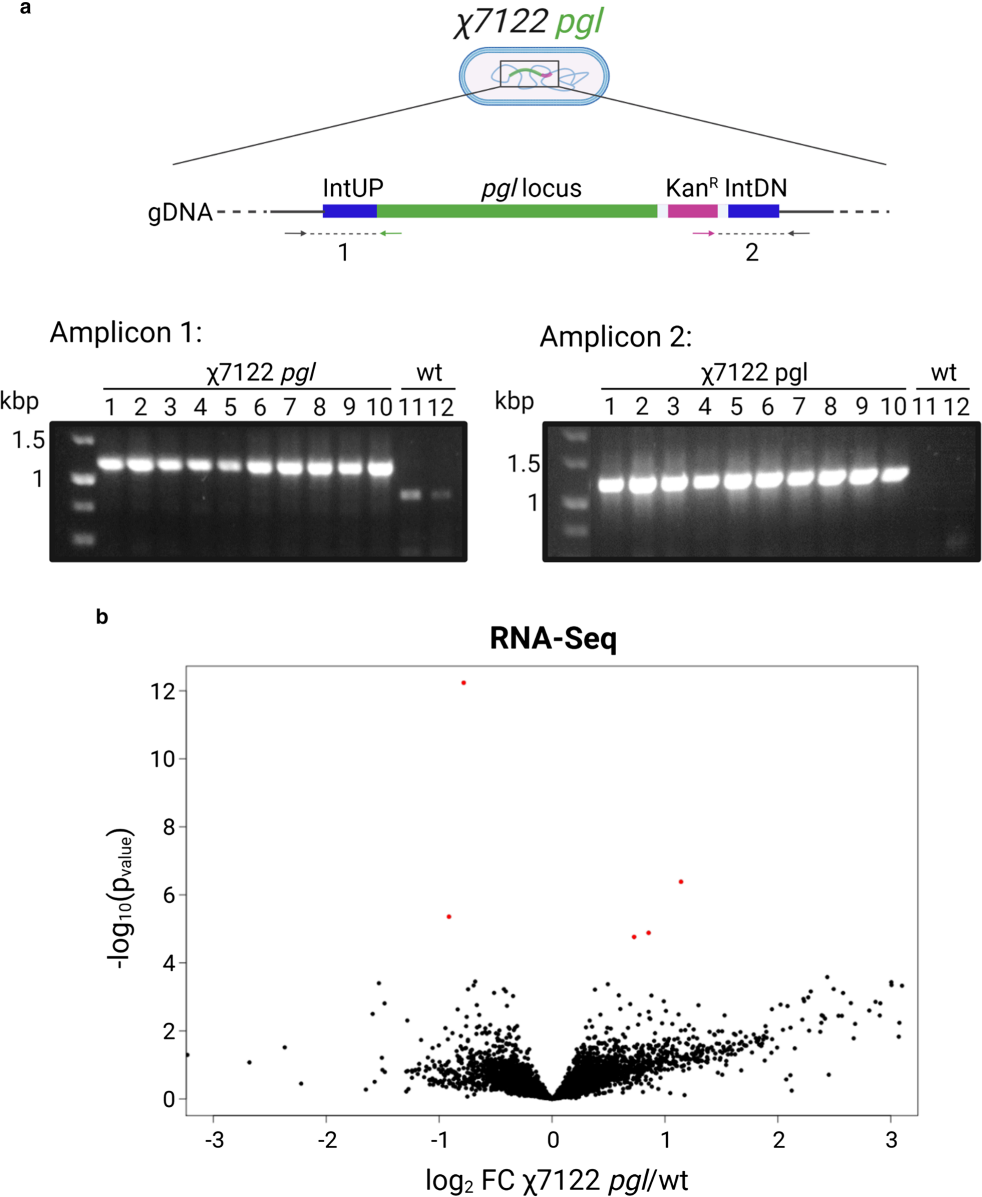
Generation of χ7122 *pgl* integrants. **a** Colony PCR confirmation of correct integration of the *pgl* locus into APEC χ7122 genome. Ten Nal^R^, Kan^R^, sucrose^R^, Amp^S^ colonies were screened (lanes 1–10), χ7122 wt (lanes 11–12) was used as a negative control. Diagnostic junction amplicons 1 and 2 (~ 1250 bp) confirm all 10 clones screened are correct. **b** Volcano plot displaying differentially expressed genes comparing χ7122 *pgl* to wt χ7122. Positive *x* axis represents up-regulation in χ7122 *pgl*. Negative *x* axis represents up-regulation in wt χ7122. Red points have adjusted p-value < 0.05 0.04% of reads from χ7122 *pgl* libraries align to the *C. jejuni pgl* locus confirming its expression at the transcriptional level. Libraries were prepared in triplicate of PCR-positive clone 2

The correctly assembled pSEC*pgl* plasmid transformed into *E. coli* S17-1 λ *pir* cells was delivered by conjugation to the recipient APEC χ7122 strain generating χ7122 *pgl* integrants. APEC χ7122 is naturally resistant to nalidixic acid (Nal^R^) [45], while the donor S17-1 λ pir strain is sensitive to it, thus the presence of this antibiotic counter-selects against the donor strain. Kanamycin resistance (Kan^R^) is conferred by the tagged *pgl* locus, therefore it is indicative of its presence in the receiver strain. Merodiploids subject to a single recombination are sucrose sensitive owing to the presence of the *sacB*-encoded levansucrase. Following amplification of merodiploids in the absence of ampicillin selection for double recombinants that are resistant to sucrose (owing to loss of pCVD422 and the endogenous copy of the target region), but which retain the kan^R^ -marked *pgl* region, were selected. To ensure plasmid backbone loss, clones were tested for ampicillin sensitivity (Amp^S^). Ten Nal^R^, Kan^R^, sucrose^R^, Amp^S^ colonies were screened by colony PCR with primers aligning to regions immediately upstream and downstream of the IntUP and IntDN homology arms, and primers aligning to either the beginning or the end of the tagged *pgl* locus (Fig. 2a, top). PCR products are amplified only if the tagged *pgl* locus has been inserted at the chosen location, thus wild-type (wt) APEC colonies were used as negative controls. Resulting PCR products (~ 1250 bp) were analysed by 1% DNA agarose gel electrophoresis and confirmed integration of the *pgl* locus at the right site in all ten clones analysed (Fig. 2a). To further confirm this and exclude off-target secondary mutations occasionally associated with use of positive-selection suicide vectors [51], both wt χ7122 and χ7122 *pgl* integrant (four clones of PCR positive colonies) were sequenced using Illumina paired end sequencing with a target coverage of 100x. The wt strain sequence showed 36 variants when compared to the published genome sequence, NZ_HE962388, while χ7122 *pgl* integrant showed 8 variants when compared to the wt (Additional file 2). Those included 6 deletions, 1 insertion and a SNP (Table 1). Six of these variants were found in intergenic region and pseudogenes, except for one found within the *flgG* gene and one within a hypothetical protein-encoding gene. No variants were found within the integrated *pgl* locus.

**Table 1:** Filtered variants of χ7122 *pgl* integrant in comparison to its wild-type counterpart

	#Chrom	Position	Reference	Alternative	Variant type	Feature
1	HE962388	472,203	CAAAAAAA	CAAAAAA	deletion	intergenic
2	HE962388	824,578	GAAAAAA	GAAAAA	deletion	pseudogene
3	HE962388	1,202,551	CGT	CT	deletion	*flgG*
4	HE962388	1,747,220	GCCC	GC	deletion	intergenic
5	HE962388	2,644,712	GAAAAAAA	GAAAAAAAA	insertion	hypothetical protein
6	HE962388	3,999,175	GA	G	deletion	intergenic
7	HE962388	4,305,775	ATTTT	ATTT	deletion	intergenic
8	HE962388	4,305,829	G	A	SNP	intergenic

RNA-Sequencing was then performed to understand whether the insertion of *C. jejuni pgl* locus in χ7122 alters the transcriptome. RNA-Seq libraries were prepared in triplicates from wt χ7122 cells and χ7122 *pgl* cells. Differential gene expression analysis indicated that the chromosomal insertion of the *pgl* locus does not significantly perturb the χ7122 transcriptome, as most of the differences observed in transcript expression levels are not significant (Fig. 2b). Where differences were detected, transcripts in χ7122 *pgl* were mostly upregulated in comparison to χ7122 wt, suggesting the introduction of a heterologous glycosylation pathway only modestly alters host strain metabolism (Fig. 2b). Five transcripts demonstrated an adjusted *p-*value < 0.05 (shown in red), suggesting statistically significant levels of differential gene expression when comparing χ7122 *pgl* and wt strains. Two transcripts—*ycbC* and *gidA*—had greater levels of expression in wt χ7122, whereas three—*ygeV, folk* and *ycfC*-demonstrated greater levels of expression in χ7122 *pgl* integrants. Gene ontology analysis revealed no link between the biological function of these genes. Read alignment also confirmed transcription of the *pgl* locus, with 0.04% of reads aligning to genes located within the *pgl* locus (Additional file 1: Table S1).

### Functional testing of χ7122 *pgl* integrants

**Fig. 3:**
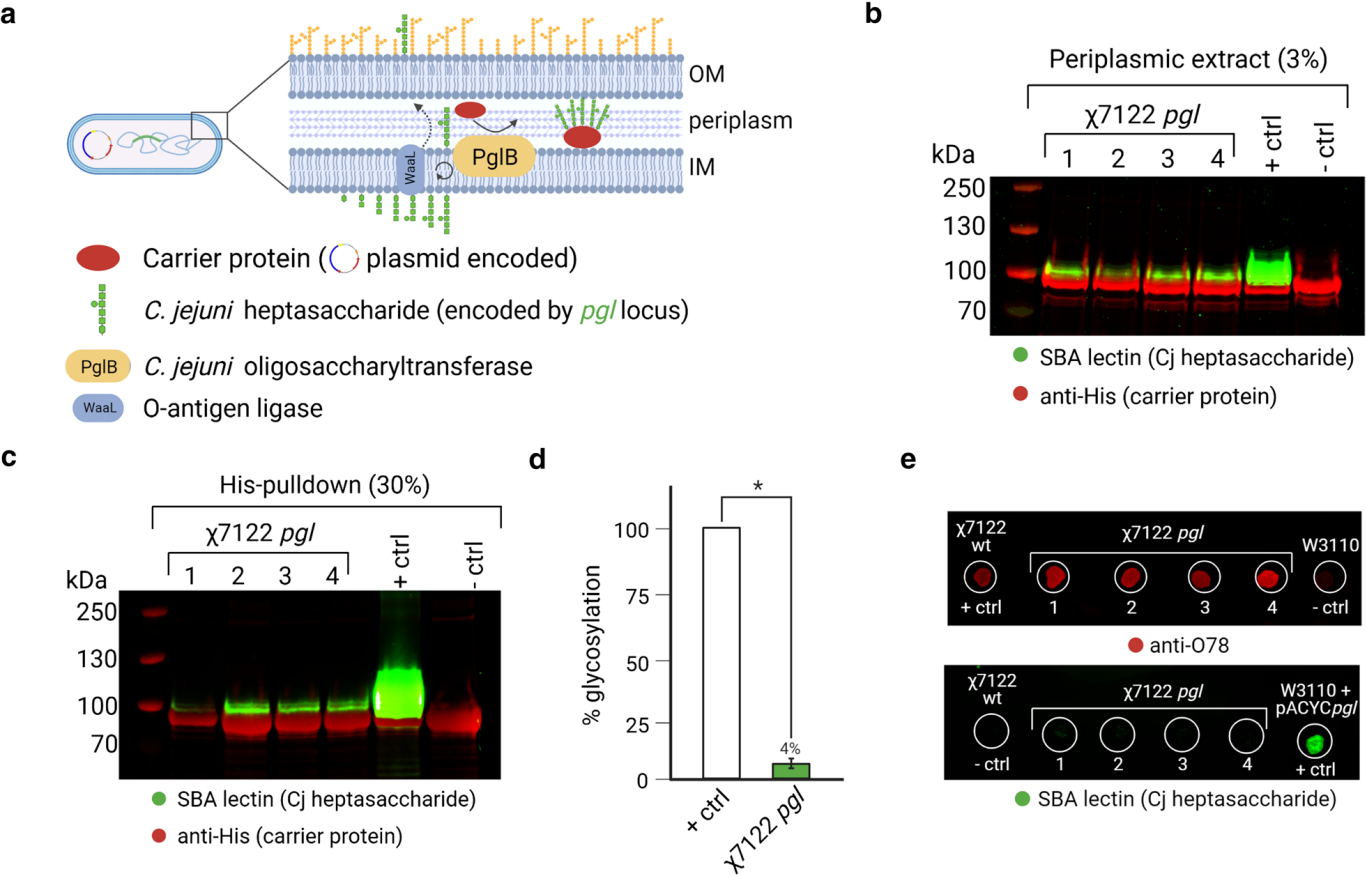
χ7122 *pgl* integrants express a functional *N*-glycosylation system. **a** Schematic representation of an χ7122 *pgl* integrant and its function; **b** western blotting of OD_600nm_ normalised periplasmic extracts showing the four χ7122 *pgl* clones tested are capable of decorating a heavily glycosylatable version of acceptor protein G-ExoA(10) with *C. jejuni* heptasaccharide, + ctrl consists of glycoengineering *E. coli* strain SDB1 transformed with a plasmid encoding the *pgl* locus (pACYC*pgl*) and a plasmid expressing L-arabinose inducible G-ExoA(10) (this latter present in all other samples);− ctrl consists of χ7122 wt transformed with G-ExoA(10)-encoding plasmid. Percentage in parenthesis indicates sample loading (v/v) per lane; **c** western blotting showing results of a His-pulldown from clarified lysates of the same samples shown in **b**; **d** densitometry analysis of **b** indicate χ7122 *pgl* integrants achieve ~ 4% glycosylation efficiency of G-ExoA(10) in comparison to + ctrl, * indicates p < 0.05 (two-tailed t-test); **e** dot-blotting of OD_600nm_ normalised cells washed and resuspended in PBS shows χ7122 *pgl* integrants maintain surface expression of their own O78 O-antigen, while no detectable surface expression of *C. jejuni* glycan is observed. + ctrl and – ctrl for O78-antigen detection are χ7122 wt and O-antigen negative *E. coli* W3110, respectively. + ctrl and − ctrl for *C. jejuni* glycan detection are W3110 constitutively expressing the *pgl* locus from pACYC plasmid and χ7122 wt, respectively. The four clones of χ7122 *pgl* tested are those verified by sequencing

After having confirmed integration of the intact *pgl* locus at the correct site, and its expression at the transcriptional level, the next step was to confirm functionality of the integrated *pgl* locus. If functional, χ7122 *pgl* integrants would now be able to synthesize all the enzymes needed for the sequential assembly of *C. jejuni* heptasaccharide (GalNAc-α1,4-GalNAc-α1,4-[Glc-β-1,3]GalNAc-α1,4-GalNAc-α1,4-GalNAc-α1,3-diNAcBac-β-1, where GalNAc is N-acetylgalactosamine, Glc is glucose, and diNAcBac, a bacillosamine derivative, is 2,4-diacetamido-2,4,6-trideoxyglucopyronose) onto the lipid carrier undecaprenyl-pyrophosphate exposed to the cytoplasmic side of the inner membrane [52]. After being flipped to the periplasmic side of the inner membrane [53], the undecaprenol-linked heptasaccharide would form a substrate for the OST PglB, which transfers it to an asparagine residue (N) within the glycosylation sequon D/EXNXS/T (where X is any amino acid except proline) in the acceptor protein [26], and theoretically for the endogenous *E. coli* O-antigen ligase WaaL, which has relaxed specificity for its polysaccharides substrates [54]. A schematic representation of a functional χ7122 *pgl* integrant is shown in Fig. 3a.

Firstly, to determine whether APEC χ7122 cells are capable of glycosylating acceptor proteins with the *C. jejuni* heptasaccharide, wt χ7122 strain was transformed with plasmid pACYC*pgl* (~ 10–12 copies per cell) constitutively expressing the *pgl* locus [24] and with pEC415 plasmids expressing glycosylatable versions of *Pseudomonas aeruginosa* ExoA toxoid, referred to as G-ExoA, upon L-arabinose induction. Acceptor protein G-ExoA carrying either two, G-ExoA(2) or ten, G-ExoA(10) glycosylation sequons were chosen as they have been used extensively as carriers and as they are well expressed and soluble in periplasmic fractions enabling ease of glycoconjugate detection [20, 55, 56]. χ7122 with plasmid-encoded *pgl* was able to *N*-glycosylate both versions of G-ExoA indicating functionality of the *pgl* locus in a pathogenic strain of *E. coli* (Additional file 1: Figure S1). Notably, glycosylation of G-ExoA(10) was more efficient and more easily detectable by SDS-PAGE followed by western blotting, thus this heavily glycosylatable version was chosen to test functionality of χ7122 *pgl* integrants bearing a single copy of the *pgl* locus.

χ7122 *pgl* integrants were transformed with the plasmid encoding inducible G-ExoA(10). Carrier protein expression and glycosylation were tested under different culture conditions, which led to the identification of variables that favour protein glycosylation in APEC χ7122 (Additional file 1: Figure S2). The expression of G-ExoA(10) was induced with L-arabinose at OD_600nm_ ~ 0.8. Cultures were grown overnight (ON) at 28 °C and G-ExoA(10) was purified the next day via periplasmic extraction or via His-pulldown from clarified whole cell lysates (WCL) followed by size separation by SDS-PAGE and western blotting. A soybean agglutinin (SBA) lectin was used to detect *C. jejuni* heptasaccharide as it binds to glycans terminating with GalNAc residues, and an anti-His antibody was used to detect C-terminally His-tagged acceptor proteins. Western blotting results demonstrate all four χ7122 *pgl* integrants tested are capable of decorating G-ExoA(10) with the *C.jejuni* glycan (Fig. 3b and c). However, the efficiency of χ7122 *pgl* in glycosylating the acceptor protein G-ExoA(10) amounts to only 4% of the positive control (+ ctrl) consisting of glycoengineering strain SDB1 expressing the *pgl* locus from pACYC*pgl* (Fig. 3d and Additional file 1: Figure S3). This difference is likely due to a combination of factors, including higher expression of the *pgl* locus from the pACYC*pgl* plasmid, more favourable glycan to protein coupling by PglB in SDB1 due to the lack of competition between different lipid-linked glycan substrates for PglB and WaaL, and possibly a lower metabolic burden as SDB1 is a W3110-derivative of *E. coli* K-12 lacking its own O-antigen, the O-antigen ligase WaaL and the enterobacterial common antigen (ECA) initiating transferase WecA saving the cells the energy investment required to produce and ligate endogenous glycans [57].

Next, we interrogated whether χ7122 *pgl* could couple the heptasaccharide to the lipid A core via the endogenous WaaL ligase. χ7122 *pgl*, χ7122 wt and *E. coli* K-12 W3110 were cultured, harvested, washed and resuspended in optically transparent phosphate-buffered saline (PBS) to test surface exposure of the glycan antigen via dot-blotting. All χ7122 *pgl* integrants showed expression and surface exposure of their own O78 O-antigen, while no surface-exposed heptasaccharide was detected (Fig. 3e). O-antigen negative *E. coli* W3110 was used as a negative control for O78-antigen detection, and W3110 with pACYC*pgl* was used as a positive control for surface exposure of the lipidA-linked heptasaccharide.

### Assessing the in vivo fitness of χ7122 *pgl* integrants

**Fig. 4:**
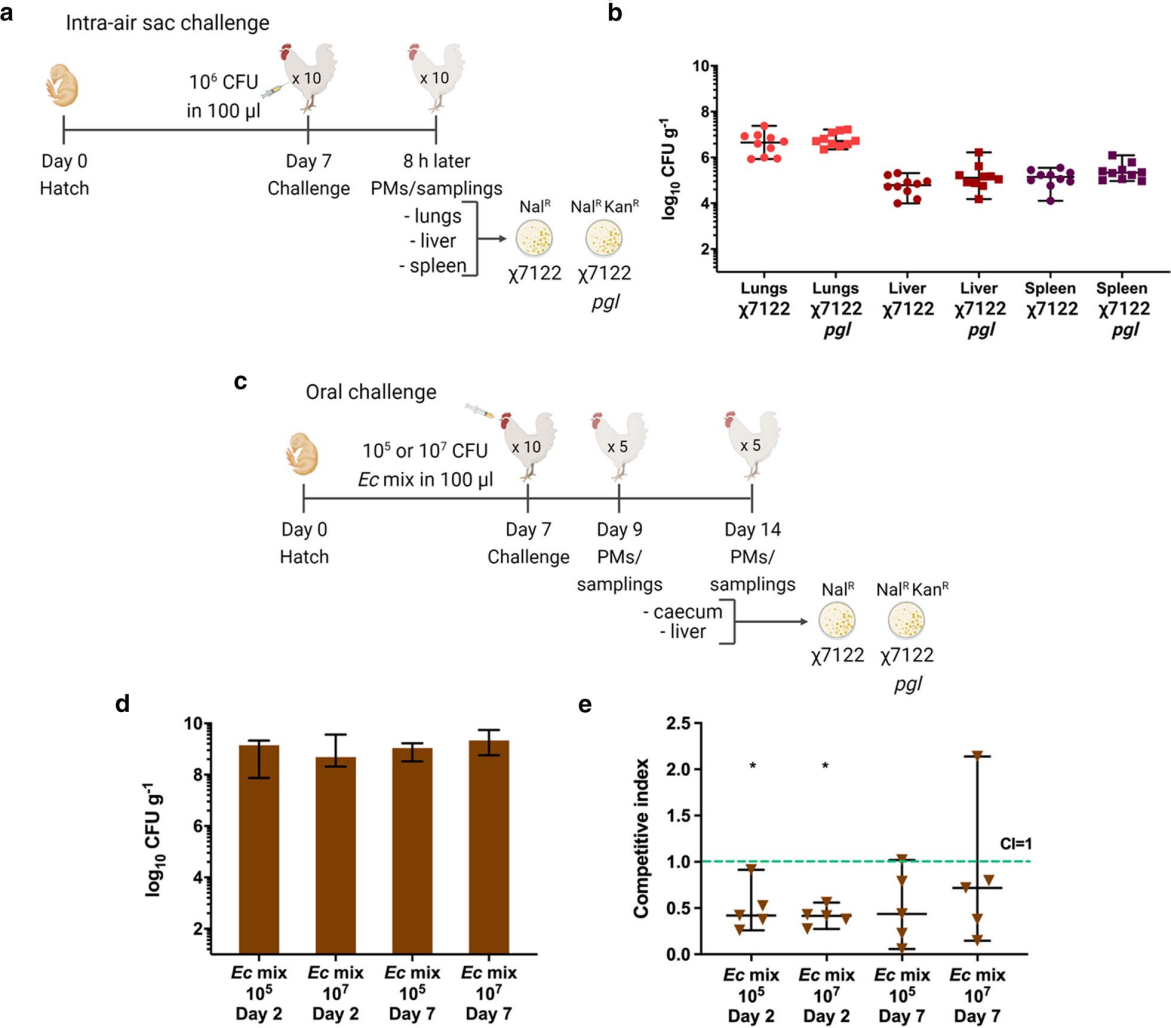
In vivo fitness and virulence of a χ7122 *pgl* integrant following intra-air sac or oral challenge of chickens. **a** Experimental design of intra-air sac challenge model; **b** Colonisation levels of χ7122 wt and χ7122 *pgl* in chicken lungs, liver and spleen were assessed 8 h post challenge; **c** Experimental design of oral challenge model; **d** Colonisation levels of mixed cultures of χ7122 wt and χ7122 *pgl* observed 2 days and 7 days post-challenge with either 10^5^ or 10^7^ challenge doses in the caecum. Colonisation levels are expressed as log_10_ CFU/g; **e** Competitive indices of χ7122 wt and χ7122 *pgl* in the caecum observed 2 and 7 days post-challenge with either 10^5^ or 10^7^ challenge doses. Statistically significant differences are highlighted with * indicating p < 0.05 (Mann–Whitney U test). Data are presented as median values with 95% confidence intervals

To establish whether the heterologous expression of *C. jejuni pgl* locus in APEC χ7122 has a fitness cost, we assessed phenotypes of the sequenced χ7122 *pgl* integrant in vivo. Groups of 10 chickens were challenged with wt χ7122 and χ7122 *pgl* either individually via the intra-air sac route or in a 1:1 mixture of wt χ7122: χ7122 *pgl* orally. The challenge dose amounted to 3 × 10^6^ CFU resuspended in medium for intra-air sac delivery, and 1.5 × 10^5^ or 1.5 × 10^7^ CFU for oral delivery (Fig. 4a and c). In the intra-air sac challenge model, birds were humanely culled 8 h post-challenge as they exhibited moderate to severe signs of infection consistent with previous research using χ7122. Both χ7122 wt and χ7122 *pgl* colonised the lungs at similar levels of 4.49 × 10^6^ and 5.2 × 10^6^ CFU/g, respectively. As typical following intra-air sac inoculation with this strain, translocation to the liver and spleen was detected, but there was no significant difference in the colonisation of these tissues between strains (Fig. 4b). In the oral challenge model, robust colonisation of the caeca was observed at days 2 and 7 post-challenge in chickens challenged with both doses of mixed cultures of χ7122 wt and χ7122 *pgl* (*Ec* mix) with median total bacterial counts ranging from 4.8 × 10^8^ to 2.2 × 10^9^ CFU/g (Fig. 4d). From the competitive indices, where an index of 1 indicates equal fitness between χ7122 wt and χ7122 *pgl*; χ7122 *pgl* was found to be significantly attenuated at day 2 post-oral challenge but not at day 7 for both challenge doses tested (Fig. 4e). Livers were colonised very poorly. If bacteria were detected at all, the χ7122 *pgl* integrant was found in substantially lower numbers than the wt (data not shown). These results indicate modest attenuation of the χ7122 *pgl* integrant as compared to wt χ7122. Nevertheless, χ7122 *pgl* persists in the caecum for at least 7 days and appeared virulent via the respiratory route. It is acknowledged that attenuating mutations are likely to be needed to make an χ7122 *pgl* integrant suitable for field use, as for example with the *aroA* mutation in a current χ7122 O78 commercial vaccine [58]. However, as the χ7122 *pgl* strain colonises the chicken intestine to high levels in the absence of overt pathology it was deemed suitable for pilot studies to determine if the vectored heptasaccharide glycan is protective against *C. jejuni* colonisation.

### Generation of a trivalent live vaccine candidate

**Fig. 5:**
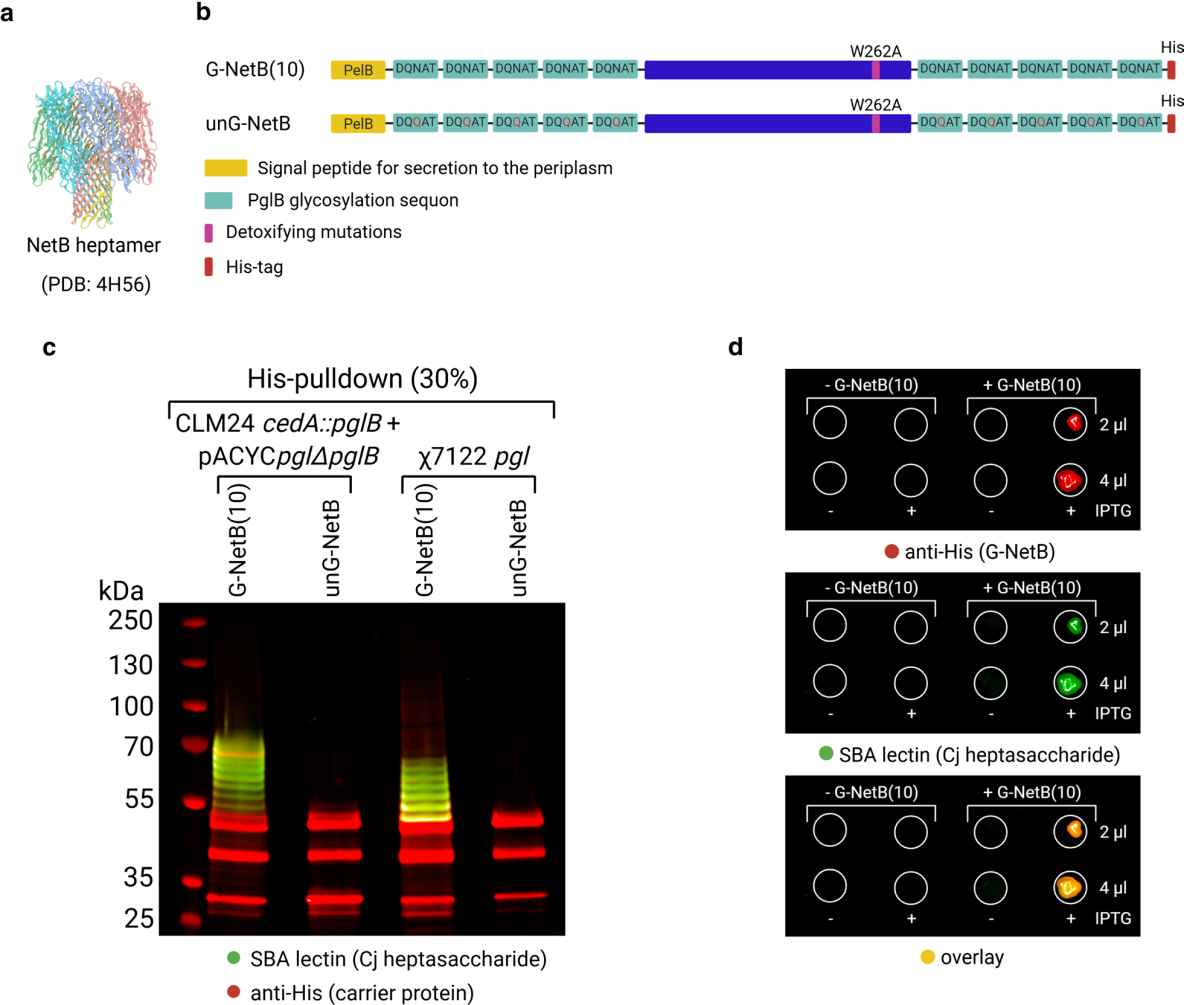
In vitro characterisation of a NetB-*C. jejuni* heptasaccharide glycan conjugate expressed from an inducible promoter in *E. coli* CLM24 *cedA::pglB* strain and χ7122 *pgl*. **a** Crystal structure of pore-forming toxin NetB in its heptameric form (PDB: 4H56) [61]; **b** Schematic linear structures of NetB monomers modified to be suitable PglB substrates. G-NetB(10) is a glycosylatable toxoid version N-terminally fused to a PelB leader peptide for translocation to the periplasm, containing ten glycosylation sequons, and a C-terminal 6xHis tag. unG-NetB is the non-glycosylatable counterpart where each sequon has a NQ mutation abrogating N-linked glycosylation; **c** Western blotting showing results of a His-pulldown of G-NetB(10) and unG-NetB from *E. coli* glycoengineering strain CLM24 *cedA::pglB* containing a chromosomal copy of PglB and a plasmid encoding the *pgl* genes necessary to produce *C. jejuni* heptasaccharide (pACYC*pgl*Δ*pglB*) in lanes 1–2, and χ7122 *pgl* integrant in lanes 3–4. Percentage in parenthesis indicates sample loading (v/v) per lane; **d** dot-blot of OD_600nm_ normalised cells washed and resuspended in PBS shows G-NetB(10) glycoconjugate is exposed on the cell surface in CLM24 *cedA::pglB*, O-antigen negative and WaaL negative glycoengineering strain carrying pACYC*pgl*Δ*pglB*. IPTG induction that promotes the expression of both PglB and G-NetB(10) in this strain is indicated with + , if present, or −, if absent

Having proven that χ7122 *pgl* is proficient in chicken gut colonisation without inducing colibacillosis, the next decision was which carrier protein we should select as an acceptor for the *C. jejuni* heptasaccharide glycan. Important attributes of vaccine antigens are immunogenicity and surface exposure, thus we sought to identify a protein relevant for poultry pathology that would meet these criteria. In this way, the protein would not only act as a carrier for the presentation of the glycan antigen but would also act as an immunological trigger and protective antigen per se. Necrotic enteritis toxin B-like (NetB) from *C. perfringens* was identified as a suitable candidate. NetB is a key virulence factor of *C. perfringens* associated with severe outcomes of necrotic enteritis in chickens [59], a disease characterised by a sudden depression followed by a rapid increase in flock mortality that poses a significant threat to the poultry industry [60]. The 3D crystal structure of NetB (Fig. 5a) revealed that NetB assembles as a heptamer to produce a pore-forming toxin capable of inserting itself in the membranes of target cells [61]. The authors of the study also identified a single amino acid mutation (W262A) that detoxifies the protein while preserving its ability to form the membrane-inserting heptamer [61], providing a useful construct well qualified for a subunit vaccine. Importantly, surface expression of NetB has been demonstrated in the host bacterium *C. perfringens*, and a *Salmonella*-vectored vaccine expressing NetB showed partial protection in a chicken model [44]. We modified the detoxified protein by adding a PelB leader signal at the N-terminus for transport to the periplasm of Gram-negative bacteria and two or ten consensus glycosylation sequons (DQNAT), rendering the protein a PglB substrate suitable for use with PGCT. We refer to these glycosylatable versions of the protein as G-NetB(2) and G-NetB(10) (Additional file 1: Figure S4a). Similarly to G-ExoA(10), G-NetB(10) showed considerably better glycosylation than G-NetB(2) in *E. coli* K-12 glycoengineering strains as well as in χ7122 *pgl* integrants (Additional file 1: Figure S4b). Interestingly, the addition of ten glycosylation sequons improved significantly the solubility of the protein, thus subsequent work was performed solely with G-NetB(10). As a control, an unglycosylatable version, unG-NetB, was designed in which the glycan acceptor asparagine residues were mutated synonymously to glutamine residues (DQNAT DQQAT) as shown in Fig. 5b. Both G-NetB(10) and unG-NetB were cloned into a pEXT20 backbone [62] where a P_tac_ promoter drives their high-level expression upon IPTG-mediated induction. Expression of G-NetB(10) and unG-NetB in the glycoengineering strain CLM24 *cedA::pglB*, carrying a chromosomal copy of IPTG-inducible PglB and using genes encoded by pACYC*pgl*Δ*pglB* for glycan assembly, showed glycosylation of the G-NetB(10) acceptor protein with eight individual bands stained by SBA lectin indicating glycosylation of G-NetB(10) at one to eight sites (Fig. 5c, lane 1). UnG-NetB exhibited no glycosylation serving as a negative control (Fig. 5c, lane 2 and 4). To our surprise, the χ7122 *pgl* integrant also efficiently glycosylated G-NetB(10) to a similar extent as the CLM24 *cedA::pglB* strain (Fig. 5c, lanes 3). To understand if G-NetB(10) glycoprotein localises to the surface of *E. coli* cells, a dot-blot of PBS-washed O-antigen negative and WaaL negative *E. coli* cells expressing glycosylated G-NetB was performed, suggesting surface exposure of the glycoprotein (Fig. 5d). The absence of the O-antigen and its ligase, WaaL, ensures the protein signal (anti-His) and glycan signal (SBA lectin) observed on the cell surface are solely due to exposure of G-NetB decorated with *C. jejuni* heptasaccharide. The CLM24 *cedA::pglB* glycoengineering strain was chosen for this experiment as its performance in glycosylating G-NetB was superior to W3110-derivative SDB1. Efficient glycosylation of G-NetB(10) by χ7122 *pgl* and surface exposure of this glycoprotein reinforced the choice of NetB as a carrier protein for our candidate trivalent vaccine with the potential to reduce APEC, *Campylobacter* and *C. perfringens* infections.

**Fig. 6:**
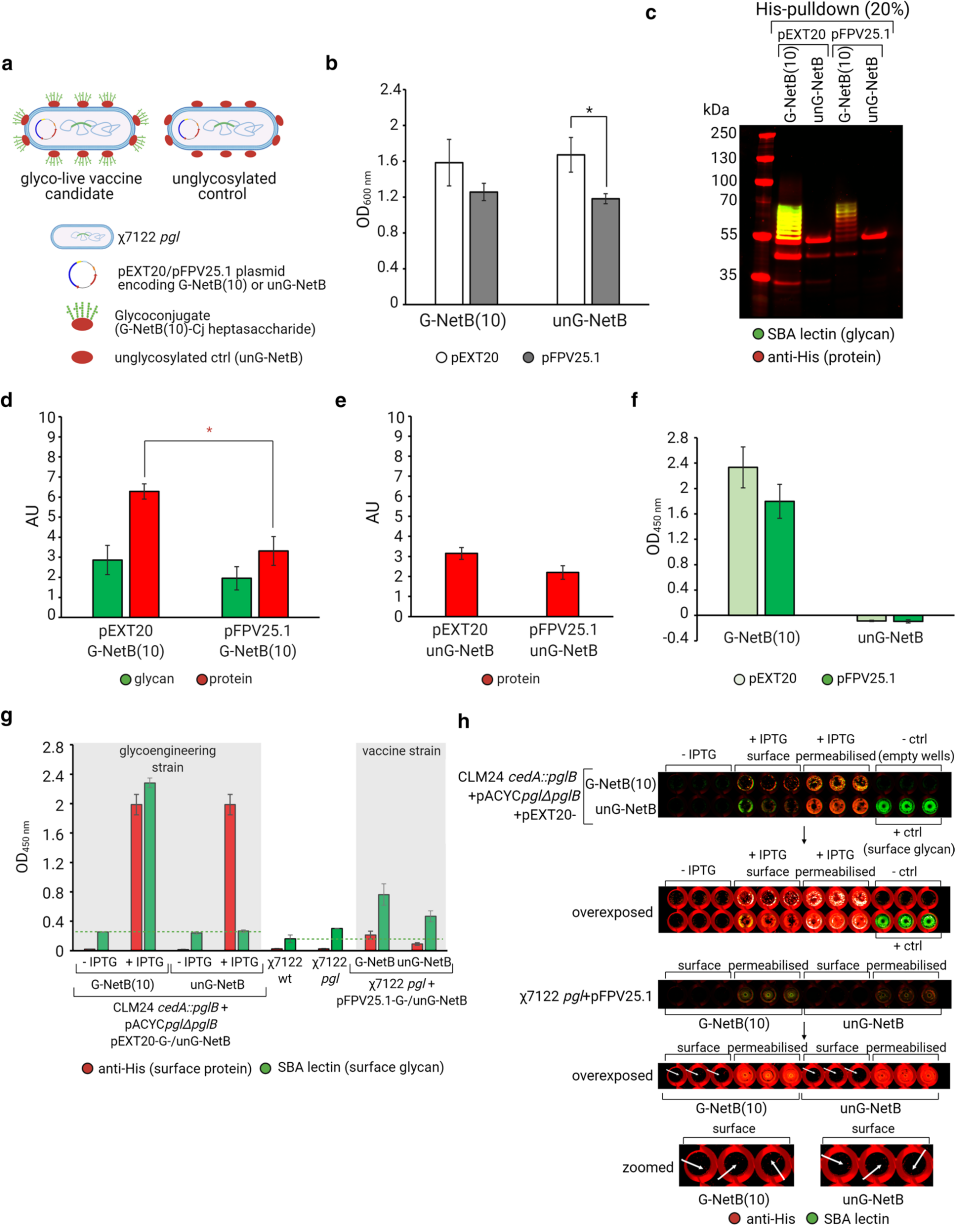
Analysis of the glycoconjugate vaccine candidate in vitro. **a** Schematic representation of the glycoconjugate vaccine candidate and its unglycosylated counterpart serving as a negative control; **b** Estimated biomass of vaccine strains after overnight culture at 28 °C, values plotted are OD_600nm_ means ± standard deviations of triplicates; **c** SDS-PAGE followed by western blotting of His-purified G-NetB(10) and unG-NetB expressed in χ7122 *pgl* from either IPTG-inducible pEXT20 or constitutive pFPV25.1 backbone, percentage in parenthesis indicates sample loading (v/v) per lane; **d**, **e** semi-quantitative densitometry analysis of glycan and protein content of G-NetB(10) glycoconjugates (**d**) and unG-NetB control (**e**), values plotted are OD-normalised mean intensities ± standard deviations of triplicates (see western blots in Additional file 1: Figure S5 for details), AU, arbitrary units; **f** Sandwich ELISA on purified glycoconjugate G-NetB(10) and unG-NetB control to relatively quantify the levels of heptasaccharide coupled to G-NetB(10) carrier expressed from the χ7122 *pgl* strains. **g** Whole cell ELISA on the CLM24 *cedA::pglB* glycoengineering strain and χ7122 *pgl* vaccine strains to relatively quantify the surface display of G-NetB(10) and unG-NetB. IPTG was used to induce NetB expression from pEXT20 vector in the glycoengineering strain, NetB expression from pFPV25.1 in the vaccine strains is instead constitutive. The green dotted line represents a non-specific signal detected when staining CLM24 *cedA::pglB* + pACYC*pglΔpglB* (left) and χ7122 wt cells (right) with SBA lectin. Values plotted in (f-g) are background subtracted OD_450nm_ means ± standard deviations of three biological replicates. Background is set to zero, glycosylation signal of unG-NetB constructs was below background. Panels c-f are different analysis of the same set of triplicates. Statistically significant differences are highlighted with * indicating p < 0.05 (two-tailed t-test). **h** OCW on CLM24 *cedA::pglB* glycoengineering strain and χ7122 *pgl* vaccine strains to visually detect the surface display of G-NetB(10) and unG-NetB. Cell permeabilization was performed to detect NetB antigens localised into the periplasm. IPTG was used to induce NetB expression from pEXT20 vector in the glycoengineering strain, while NetB expression from pFPV25.1 in the vaccine strains is constitutive. Empty wells stained with both primary and secondary antibodies were used as negative controls (− ctrl), while O-antigen negative, WaaL positive *E. coli* cells expressing pSEC*pgl* were used as positive controls (+ ctrl) for surface display of the heptasaccharide (lipid-linked surface glycan)

While inducible expression of G-NetB(10) from pEXT20 will ensure that the glycoprotein is present at the point of vaccination, it is not certain that the promoter would be active in vivo in the absence of IPTG or endogenous lactose. To drive the constitutive expression of G-NetB(10) and unG-NetB during colonisation of chickens, the two constructs were cloned into the pFPV25.1 vector under the control of the *S.* Typhimurium promoter P_rpsM_ [63]. The promoter has been proven to be active in APEC during respiratory infections [64] and in *S*. Typhimurium in the avian gut by driving the expression of fluorescent reporter proteins [65]. Performances of the constructs for inducible and constitutive expression of G-NetB(10) and unG-NetB were then compared in the χ7122 *pgl* integrant (Fig. 6a). Biomasses, measured as optical density at 600 nm (OD_600nm_), of χ7122 *pgl* cultures grown overnight showed that χ7122 *pgl* expressing IPTG-inducible G-NetB(10)/unG-NetB from pEXT20 reached similar OD_600nm_ 16 h post-induction irrespective of the presence of the glycan antigen (Fig. 6b). Likewise, the χ7122 *pgl* integrant constitutively expressing G-NetB(10)/unG-NetB from pFPV25.1 reached similar cell biomass after overnight culture, but their final OD_600nm_ were ~ 1.4 fold lower than χ7122 *pgl* expressing NetB from pEXT20 vectors, suggesting constitutive expression of NetB may be more burdensome for the cells (Fig. 6b). To verify the extent of glycoconjugate expression, cultures were harvested, lysed and glycoconjugates were purified via a His-pulldown followed by SDS-PAGE and western blotting (Fig. 6c). Both pEXT20 and pFPV25.1 vector drove expression of G-NetB(10) and unG-NetB in χ7122 *pgl,* where G-NetB(10) was successfully glycosylated. OD-normalised densitometry analysis of His-purified G-NetB(10) and unG-NetB triplicates of the samples shown in Fig. 6b indicate that the extent to which G-NetB(10) glycoconjugate is coupled to the *C. jejuni* heptasaccharide is not significantly different when NetB expression is driven by either vector (Fig. 6d). Yet, the protein content of the glycoconjugate is ~ 1.9 fold lower when expression is achieved from pFPV25.1 rather than from pEXT20 vector (Fig. 6d), where constitutive expression of G-NetB(10) seems to favour formation of a more heavily glycosylated protein (higher glycan/protein ratio, see upward shift on blot in Fig. 6c, and Additional file 1: Figure S5). The difference in protein expression is less pronounced for unG-NetB (Fig. 6e). A sandwich ELISA was performed on all purified G-NetB(10) glycoconjugates and unG-NetB controls to assess protein glycosylation levels with a technique other than densitometry. As ELISAs are highly sensitive assays, smaller difference in glycan coupling could be detected with this method. ELISA results confirmed that G-NetB(10) glycosylation is achieved from either construct to a comparable extent. To understand whether the NetB antigens localise to the surface of the χ7122 *pgl* vaccine strain an ELISA on whole cells washed and resuspended in PBS was performed (Fig. 6g). The results indicate that the χ7122 *pgl* vaccine strains are capable of localising G-/unG-NetB on the cell surface, albeit they do so considerably less efficiently than the glycoengineering CLM24 *cedA::pglB* strain. Surface display of the *C. jejuni* heptasaccharide depends on the exposure of the G-NetB(10) glycoprotein in CLM24 *cedA::pglB* glycoengineering strain, which is ligase deficient. Given the vaccine strain is WaaL positive, it is possible that surface display of the glycan epitope is not solely due to presentation of G-NetB(10) glycoprotein, but also in part to lipid A-linked heptasacchride justifying the glycan levels detected above background in the vaccine strain lacking NetB as well as in the strain delivering unG-NetB. On-cell western blotting (OCW) of fixed integer or permeabilised cells (Fig. 6h) showed that both G-NetB and unG-NetB are mostly localised in the periplasm of both CLM24 *cedA::pglB* strain and of the χ7122 *pgl* strain. While some copies of G-/unG-NetB are surface-exposed in CLM24 *cedA::pglB* glycoengineering strain, surface display of G-NetB glycoprotein or its unglycosylated counterpart, unG-NetB, is hardly detectable on the vaccine strain using this technique.

Evaluation of protection conferred by the trivalent vaccine candidate in poultry.

**Fig. 7:**
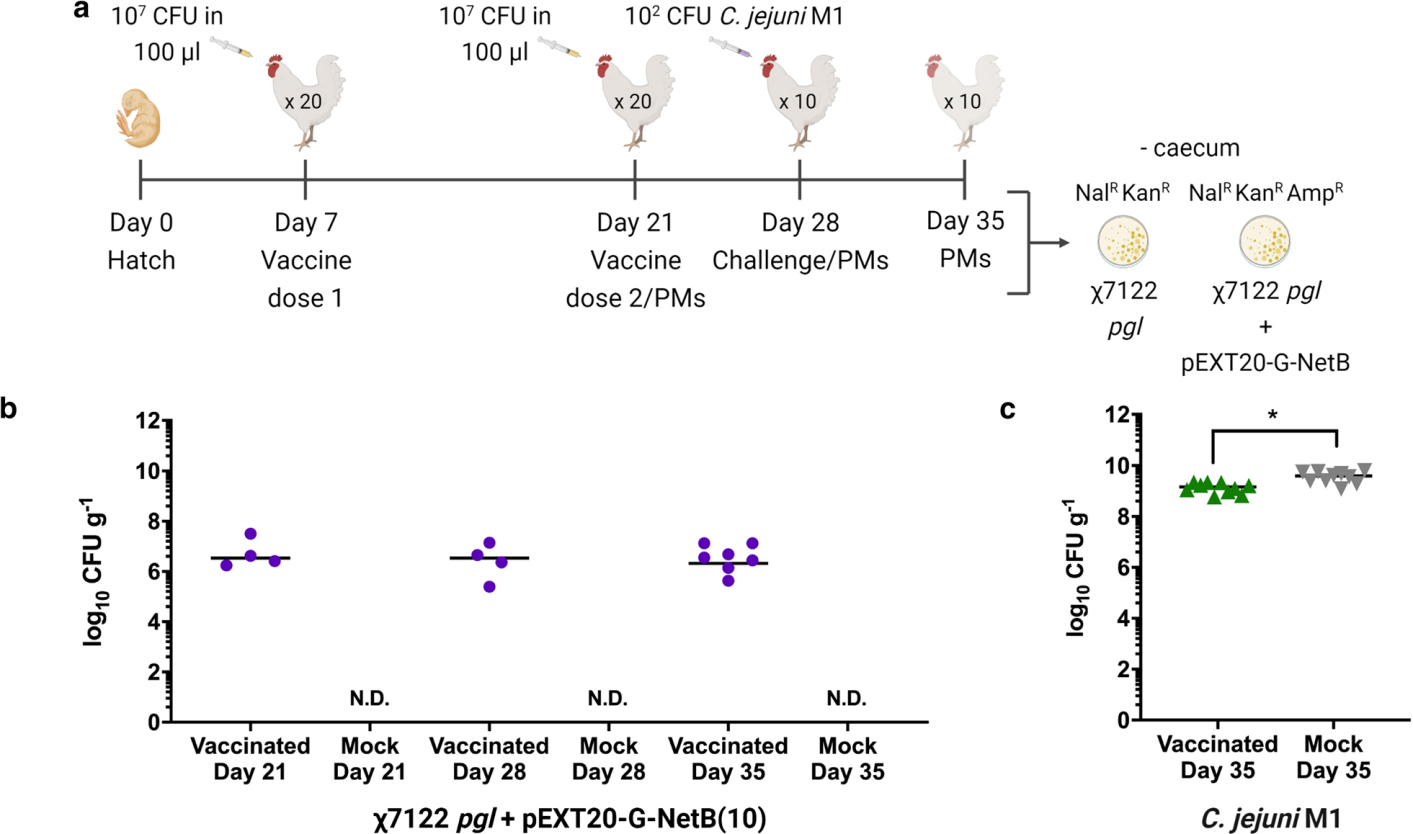
χ7122 *pgl* expressing G-NetB(10) from pEXT20 fails to substantially reduce *Campylobacter* colonisation. **a** Experimental design; **b** Strain colonisation levels in the caeca of vaccinated chickens at days 21, 28 and 35. Retrieved vaccine strains were Nal^R^, Kan^R^ only, indicating loss of Amp^R^ pEXT20-G-NetB(10) plasmid in vivo. **c** Colonisation levels of *C. jejuni* strain M1 in the caeca of vaccinated chickens one week after challenge. Colonisation levels of the caecum from either the live vaccine (**b**) or *C. jejuni* M1 (**c**) are expressed as log_10_ CFU/g. Statistically significant reductions are highlighted with * indicating p-values < 0.05 (Mann–Whitney U test). Data are represented as median values with 95% confidence intervals

We first conducted a trial with the χ7122 *pgl* integrant expressing G-NetB(10) following IPTG induction from the pEXT20 vector as shown in Fig. 7. Groups of 20 chickens were orally vaccinated at day 7 and day 21 post-hatch with 10^7^ CFU of the vaccine strain resuspended in LB medium (Fig. 7a). Mock-vaccinated birds were orally given LB medium only. At days 21 and 28, prior to second vaccination and *C. jejuni* challenge, respectively, kanamycin resistant colonies inferred to be the χ7122 *pgl* strain were isolated from the vaccinated chickens at median levels of 3 × 10^6^ CFU/g (Fig. 7b). Bacteria resistant to both kanamycin and ampicillin were not recovered (not shown), suggesting pEXT20 instability and loss in vivo. No kanamycin resistant bacteria were isolated from mock-vaccinated chickens confirming the lack of spread of the live vaccine between groups. At day 35, a week after challenge with 100 CFU of *C. jejuni* strain M1, kanamycin-resistant bacteria were recovered only from 7/10 vaccinated chickens at median levels of 2.8 × 10^6^ CFU/g (Fig. 7b). *C. jejuni* M1 was recovered from vaccinated and mock-vaccinated chickens at median levels of 1.5 × 10^9^ and 3.9 × 10^9^ CFU/g, respectively, resulting in a small, but statistically significant 0.42 log_10_ reduction in caecal carriage in the vaccinated chickens (Fig. 7c).

**Fig. 8:**
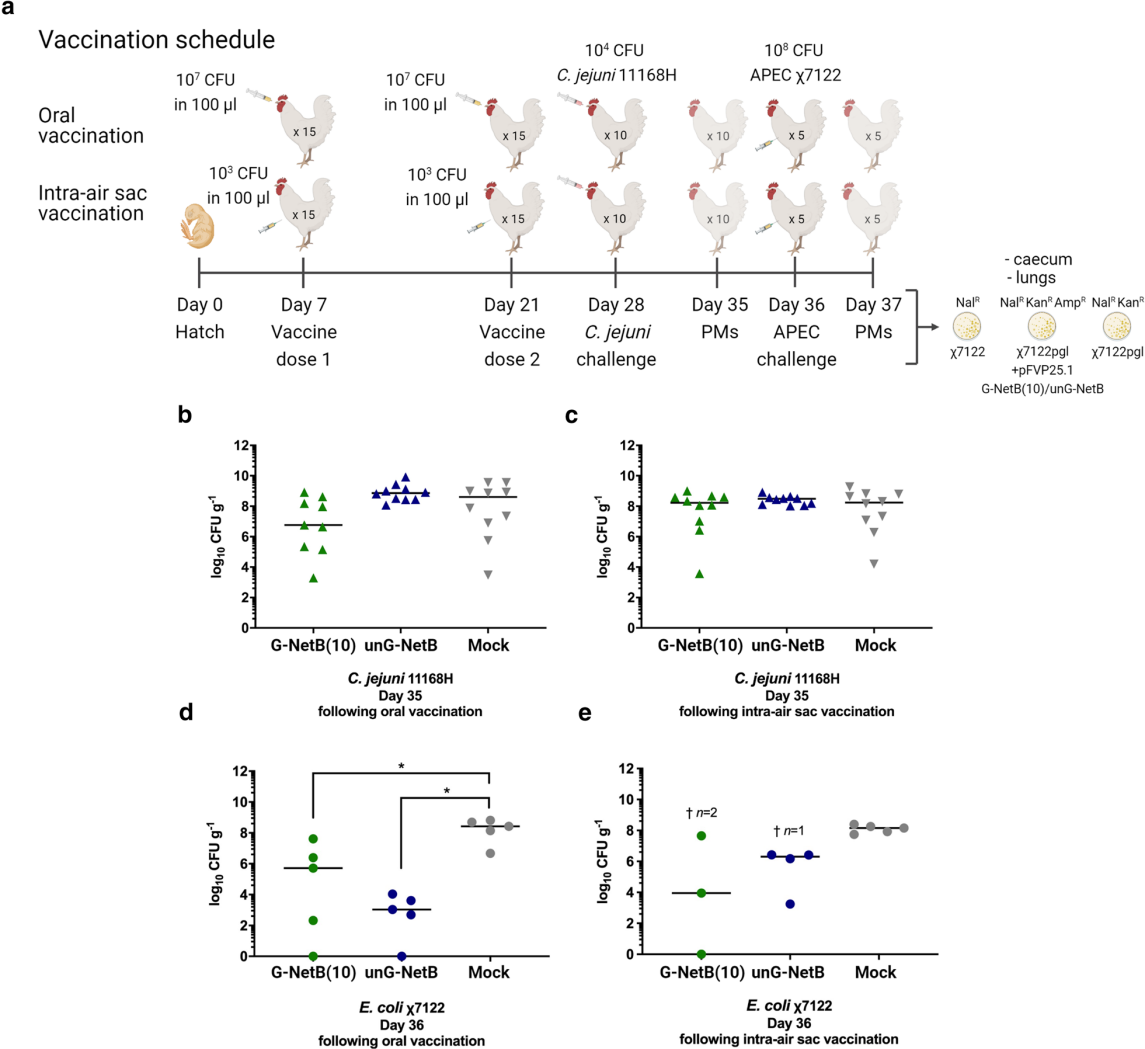
Efficacy of χ7122 *pgl* vaccine strains vectoring G-NetB(10) or unG-NetB against caecal colonisation by *C. jejuni* or lung colonisation by wt χ7122. **a** Experimental design; **b**, **c** Assessing reduction of *C. jejuni* 11168H colonisation of the caeca 1 week after challenge in **b** orally vaccinated chickens or **c** intra-air sac vaccinated chickens; **d**, **e** Assessing protection against homologous challenge with wt χ7122 in **d** orally vaccinated chickens or **e** intra-air sac vaccinated chickens. Colonisation levels of the caeca (for *C. jejuni*) and lungs (for wt χ7122) are expressed as log_10_ CFU/g. Data are represented as median values with 95% confidence intervals. Statistically significant reductions are highlighted with * indicating p < 0.05 (GLM followed by Dunn’s post hoc). † indicates animals that were euthanised on presentation of end-point criteria specified in our licence for animal research

A second trial was performed to assess protection conferred by χ7122 *pgl* constitutively expressing G-NetB(10) or its unglycosylatable control, unG-NetB, from the pFPV25.1 vector against both *C. jejuni* and APEC homologous challenge. The vaccine strains were confirmed to contain all four plasmids reported to be present in strain χ7122 by PCR for plasmid-encoded genes (Additional file 1: Figure S6) (66, 67). Upon validation, groups of 15 chickens were vaccinated orally or via the intra-air sac route at day 7 and day 21 post-hatch with 10^7^ or 10^3^ CFU of the vaccine strains, respectively (Fig. 8a). A first group was vaccinated with the G-NetB(10) expressing strain, a second group with the unG-NetB expressing strain, and a third group was mock-vaccinated with PBS only. Birds were orally challenged at day 28 with 4 × 10^5^ CFU of *C. jejuni* 11168H and at day 36 with 7 × 10^6^ CFU of wt APEC χ7122 intra-air sac. Reduction of *C. jejuni* and APEC colonisation were assessed 1 week and 1 day post challenge, respectively. Similarly to the previous trial, birds challenged with wt χ7122 were humanely culled within a day as the mock-vaccinated birds began to exhibit moderate to severe signs of infection.

Oral vaccination with χ7122 *pgl* expressing G-NetB(10) reduced mean caecal colonisation by *C. jejuni* 11168H compared to the mock-vaccinated group by nearly 2 log_10_ CFU, but the difference was not statistically significant owing to the variation in colonisation observed (Fig. 8b). No reduction was observed when the vaccine delivers unglycosylated NetB, which suggests the modest protective effect is not associated with innate immune priming or competition by the χ7122 *pgl* strain per se. By contrast, no effect on caecal colonisation by *C. jejuni* was observed following intra-air sac vaccination (Fig. 8c).

Both oral and intra-air sac vaccination with χ7122 *pgl* vaccine strain resulted in a statistically significant reduction in colonisation of the lungs by wt APEC χ7122 in comparison to mock-vaccinated chickens (Fig. 8d and e).

**Fig. 9:**
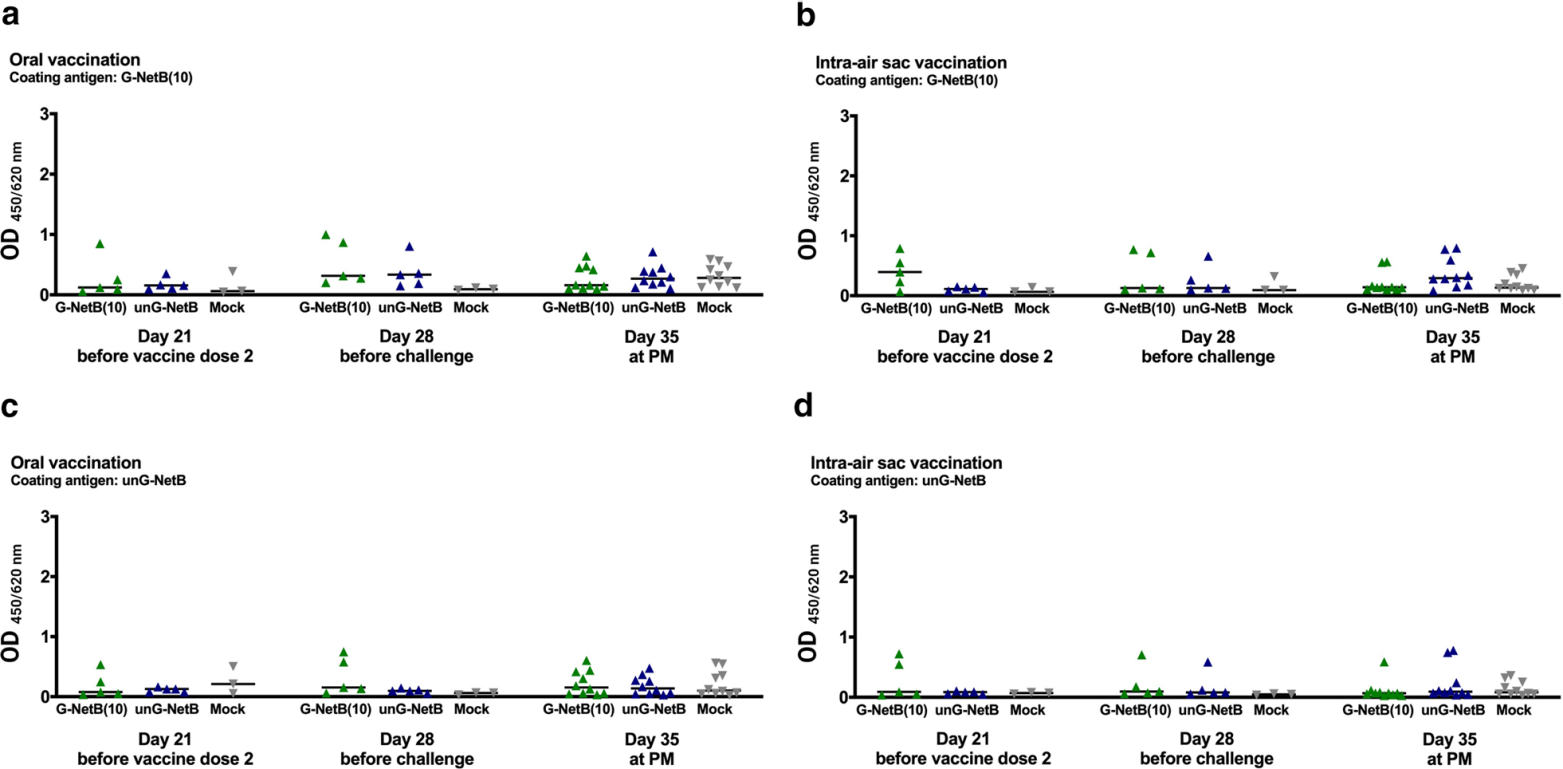
Induction of immune responses to NetB antigens following vaccination and challenge by oral and intra-air sac routes Antigen-specific serum IgY against G-NetB(10) (**a**, **b**) and unG-NetB (**c**, **d**) were measured by indirect ELISA between first and second vaccine dose (day 21), between the second vaccine dose and challenge (day 28) and at post-mortem examination (day 35). Data are represented as median values with 95% confidence intervals

To evaluate the antibody response to NetB antigens in vaccinated chickens, serological ELISA were performed using purified G-NetB(10) and unG-NetB as coating antigens (Additional file 1: Figure S7) in order to discriminate responses directed against the glycoprotein and protein-only, respectively (Fig. 9). While some birds that received χ7122 *pgl* vectoring glycosylated or unglycosylated NetB showed a moderate response relative to mock-vaccinated birds, considerable inter-animal variance was detected and consequently differences in median values proved not to be statistically different.

## Discussion

As the poultry industry grows and consumption of poultry-derived products continues to increase worldwide, prevention of infectious diseases is critical to maintain healthy flocks and to limit the spread of zoonoses from this source. Concomitantly, the rise in antimicrobial resistance is posing serious threats owing to the widespread use of antibiotics as therapeutics to control infections and as growth-promoters in farmed animals in some countries [68]. Expanding the vaccine portfolio for farmed animals is paramount for tackling this problem [69].

Veterinary vaccines need to be considerably cheaper than those for humans and easy to produce and administer at scale [70]. A biotechnology like PGCT that exploits live bacterial cells as factories to produce glycoconjugates and is applicable to pathogenic strains offers new cost-effective strategies for vaccine design well suited for the veterinary market.

In this study we presented a novel vaccine design created using PGCT. By integrating the *C. jejuni pgl* locus into the genome of an APEC strain we demonstrate that the conserved heptasaccharide antigen from *C. jejuni* can be expressed and conjugated to recombinant acceptor proteins expressed from a plasmid in a pathogenic *E. coli* strain. The introduction of a heterologous glycan synthesis pathway did not substantially alter the transcriptome Fig. 2b) or virulence following intra-air sac inoculation of chickens (Fig. 4a and b). In competition assays to study intestinal colonisation, the χ7122 *pgl* strain was slightly less fit Fig. 6 than the parent strain, but still colonised the gut effectively (Fig. 4d and f). As the strain colonises the gut in the absence of colibacillosis, it was deemed suitable for pilot studies to assess protection conferred by the vectored antigen, accepting that further attenuating mutation is likely to be required to make it suitable for field deployment. We demonstrated that a single chromosomal copy of the *pgl* locus in the χ7122 *pgl* strain efficiently glycosylates a sequon-tagged NetB similarly to an *E. coli* K-12 glycoengineering strain expressing the glycan assembly genes from a plasmid (Fig. 5c). Surface display of the antigen is limited in the χ7122 *pgl* strain (Fig. 6g and h), although the vaccine strain can still release copies of the antigen post lysis in vivo. Levels of antigen presentation are proportional to the efficacy of vaccines against the target pathogens [71], so achieving efficient glycosylation is a prerequisite for our candidate to work. This feature is very relevant for vaccines targeting *Campylobacter* spp., where differences in the glycan dose may explain differences in the efficacy of glycoconjugates tested thus far [72, 73].

We elected to express the conserved *Campylobacter* heptasaccharide encoded by the *pgl* locus in APEC, as this antigen, unlike capsular polysaccharides and O-antigens, does not vary [34]. It has a demonstrable role in *Campylobacter* spp. ability to colonise its hosts [36]. Moreover it has already shown efficacy as a vaccine antigen in recombinant glycoconjugate vaccines [41, 43]. Previously, cross-talk between bacterial glycosylation pathways has been demonstrated [74]. Since we did not affect the synthesis pathway of χ7122 endogenous glycans, such as its O-antigen and its ECA, a competition between endogenous and heterologous glycans may occur for coupling to protein acceptors and lipid anchors. We could not observe exposure of lipidA-linked heptasaccharide on the surface of χ7122 *pgl* strains by dot-blot (Fig. 3e). We speculate this could be simply due to a considerable difference in size of the two glycans, whereby the much bigger O78 O-antigen (~ 1–4 million Da) [75] could mask the lipidA-linked heptasaccharide (~ 1400 Da) [52]. Alternatively, the χ7122 WaaL ligase may largely favour transfer of its endogenous O-antigen, starting with a GlcNAc residue, over *C. jejuni* heptasaccharide, starting with a diNAcBac residue. ELISA data on whole cells suggest a minor surface display of the heptasaccharide independent of NetB glycoprotein, thus likely anchored to lipids (Fig. 6g). We also cannot exclude that structural integrity of the *C. jejuni* heptasaccharide is affected. It is possible that the heptasaccharide starts with a GlcNAc reducing end sugar, transferred by *E. coli* initiating transferase WecA, instead of bacillosamine transferred by PglC initiating transferase. However, previous evidence demonstrated that the immunogenic residues of this small heptasaccharide are the terminal ones [34], supporting the potential of our design. By coupling this glycan to an immunogenic carrier protein, we also hoped to achieve activation of both humoral and cellular immune responses, as observed in humans vaccinated with subunit glycoconjugate vaccines, thereby increasing vaccine potency and the duration of protection [14].

We tested our trivalent vaccine candidate against APEC and *C. jejuni*; testing against *C. perfringens* is yet to be performed and is complicated by the difficulty of experimentally reproducing and quantifying necrotic enteritis in chickens. The vaccine expressing glycosylated G-NetB(10) from an IPTG-inducible pEXT20 vector caused 0.42 log_10_ reduction in caeca colonisation from *C. jejuni* strain M1 (Fig. 7c). However, biologically, this reduction is unlikely to reduce transmission of *C. jejuni* to humans via the food chain. The vaccine expressing glycosylated G-NetB(10) from pFPV25.1 vector protected chickens from respiratory challenge with the homologous APEC strain (Fig. 8d, e), however reduction of colonisation with *C. jejuni* was marginal (Fig. 8b, c). Interestingly, median colonisation of the chicken caeca was reduced by 2 log_10_ CFU with our χ7122 *pgl* integrant containing the pFPV25.1-encoded G-NetB(10) vaccine in comparison with the unglycosylated control APEC *pgl* pFPV25.1 unG-NetB when delivered orally (Fig. 8b). This result is encouraging as it suggests that the decrease in colonisation is specifically due to the exposure to the *C. jejuni* glycan antigen and not to non-specific effects of the immunisation (e.g. priming of innate responses by the vector strain or direct competition between the vector and *C. jejuni*). However, the levels of reduction observed across the chickens were variable and statistical significance was not achieved. Similarly, antibody responses against the delivered antigens, G-NetB(10) or unG-NetB, were low in magnitude and not significantly higher than in the mock-vaccinated group (Fig. 9). We are currently investigating the effects of higher temperature, mimicking chicken body temperature, on the expression and glycosylation of the studied antigens as we suspect this may be a reason behind unsatisfactory protection against *Campylobacter*. A limitation of our vaccine candidate is that so far we only have evidence of protection against the homologous APEC serotype, whereas colibacillosis is known to be associated with diverse serotypes and evidence of cross-protection (and one or more attenuating mutations) would be needed for it to be commercially acceptable.

The candidate vaccine presented in this work is an innovative adaptation of PGCT using a live strain as a carrier for glycoconjugate vaccines. Modification of several APEC serotypes with the same methodology described here is possible. Moreover additional mutations to the vector strain to promote vaccine biocontainment [76], enhance bacterial lysis [77], and to boost production and surface exposure of the glycoconjugates would be advantageous and desirable for the applicability of this class of vaccines in the veterinary field.

## Conclusions

In this study we produced a novel bacterial multivalent vaccine for poultry exploiting PGCT. The vaccine is a modified APEC strain carrying a copy of *C. jejuni pgl* locus in its genome and a plasmid-encoded acceptor protein (NetB). The strain was demonstrated to be able to express *C. perfringens* toxoid NetB heavily glycosylated with the *C. jejuni* heptasaccharide antigen. The vaccine was successful in reducing colonisation of the lungs by the homologous APEC serotype and *Campylobacter* colonisation of the caeca was reduced in a glycan-specific manner albeit not statistically significantly*.* The vaccine requires further optimisation and evaluation against *C. perfringens* and *Campylobacter* in a manner that simulates natural exposure, but it paves the way for the generation of a novel class of live glycoengineered vaccines suitable for the veterinary industry.

## Methods

### Bacterial strains, plasmids and growth conditions

Electrocompetent *E. coli* strains were transformed with plasmids encoding the chosen carrier protein, and if necessary, the *C. jejuni* glycan, and PglB (glycan, carrier protein and PglB OST) in 1 mm gap cuvettes at 2 kV, 200 Ω and 25 µF. Strains were routinely grown in Lysogeny Broth (LB) or on LB agar at 37 °C with the required antibiotics at the following concentrations: 100 µg/ml ampicillin, 20 µg/ml nalidixic acid, 50 µg/ml kanamycin, 100 µg/ml trimethoprim. *E. coli* DH5α were used as a host for cloning experiments.

*C. jejuni* strains M1 and NCTC11168H were routinely cultured on charcoal-cephoperazone-deoxycholate agar (CCDA) at 40 °C under microaerophilic conditions (5% O2, 5% CO2 and 90% N2). Liquid cultures were prepared in Mueller–Hinton (MH) broth equilibrated in a microaerophilic atmosphere overnight before inoculation and incubation for 16 h with shaking at 400 rpm. For challenge of chickens, liquid cultures were adjusted based on a standard curve of CFU/ml relative to OD_600nm_, with serial dilution where required to obtain the desired challenge dose. *Inocula* used in chicken studies were confirmed by retrospective plating of tenfold serial dilutions on CCDA and determination of viable counts after incubation for 48 h.

The bacterial strains, plasmids and oligos used in this study are listed in Tables 2, 3 and Additional file 1: Table S2.

**Table 2:** Bacterial strains used in this study

Strain	Description or genotype	Reference or source
*E. coli* S17-1 λ pir	Conjugative donor strain for maintenance and transfer of the suicide *pgl* donor plasmid, pSEC*pgl*	[78]
*E. coli* χ7122	Genome-sequenced spontaneous Nal^R^ APEC O78:H9 ST23 strain isolated from the liver of a turkey with colibacillosis. Template for amplifying ~ 1 kb upstream (IntUP) and downstream (IntDN) homology arms for integration of the *pgl* locus marked with a kanamycin resistance cassette between 3,997,780 and 3,997,791 on the genome	[45]
*E. coli* χ7122 *pgl*	χ7122 *pgl* integrant carrying *C. jejuni pgl* locus marked with a kanamycin resistance cassette between position 3,997,780 and 3,997,791 on the genome	This study
*E. coli* W3110	O-antigen negative strain widely used as a safe laboratory *E. coli* K-12 strain, *wbbL*-, ƛ-, *rph-1*, IN(*rrnD-rrnE*)1	[79]
*E. coli* SDB1	W3110-derivative, Δ*waaL*, Δ*wecA*	[57]
*E. coli* CLM24 *cedA::pglB*	W3110-derivative, Δ*waaL* with genomic integration of IPTG-inducible *C. jejuni* PglB in *cedA*	Abouelhadid et al., (manuscript in preparation)
*E. coli* DH5α	Commercial strain used for cloning	New England Biolabs, #C2987H

**Table 3:** Plasmids used in this study

Plasmid	Description	Antibiotic resistance(s)	Reference or Source
pCVD442	Suicide donor plasmid based on R6K replicon and *sacB* sucrose counter selection marker	Amp	[49]
pKD4	Template plasmid for amplification of kanamycin cassette flanked with FRT sites	Kan	[80]
pEC415	Plasmid for L-arabinose inducible expression, P_ara_	Amp	[81]
pEXT20	Plasmid for IPTG inducible expression, P_tac_	Amp	[62]
pFPV25.1	Plasmid for constitutive expression, P_Rpsm_	Amp	[63]
pACYC*pgl*	Plasmid encoding *C. jejuni pgl* locus, constitutive expression	Cm	[24]
pACYC*pgl*Δ*pglB*	Plasmid encoding *C. jejuni pgl* locus depleted of OST PglB, constitutive expression	Cm, Kan	[46]
pEC415-G-ExoA(2)	L-arabinose inducible expression of His-tagged Exotoxoid A with 2 PglB glycosylation sequons under DsbA periplsmic signal	Amp	[82]
pEC415-G-ExoA(10)	L-arabinose inducible expression of His-tagged Exotoxoid A with 10 PglB glycosylation sequons under DsbA periplsmic signal	Amp	[56]
pSEC*pgl*	Suicide plasmid for genomic integration of *C. jejuni pgl* locus in APEC Χ7122 genome	Amp, Kan	This study
pEXT20-G-NetB(2)	IPTG inducible expression of His-tagged NetB toxoid with 2 PglB glycosylation sequons under PelB periplasmic signal	Amp	This study
pEXT20-G-NetB(10)	IPTG inducible expression of His-tagged NetB toxoid with 10 PglB glycosylation sequons under PelB periplasmic signal	Amp	This study
pEXT20-unG-NetB(10)	IPTG inducible expression of His-tagged NetB toxoid with 10 non-functional PglB glycosylation sequons (NQ) under PelB periplasmic signal	Amp	This study
pFPV25.1-G-NetB(10)	Constitutive expression of His-tagged NetB toxoid with 10 PglB glycosylation sequons under PelB periplasmic signal	Amp	This study
pFPV25.1-unG-NetB(10)	Constitutive expression of His-tagged NetB toxoid with 10 non-functional PglB glycosylation sequons (NQ) under PelB periplasmic signal	Amp	This study

### Plasmid cloning

#### Construction of pSECpgl plasmid

The suicide donor plasmid, pSEC*pgl*, carrying the *C. jejuni pgl* operon for integration into the genome of *E. coli* χ7122 was constructed by seven fragment assembly into the pCVD422 vector using the Gibson method. Briefly, the pCVD442 vector backbone, IntUP and IntDN (two ~ 1 kb homology regions for integration into the *E. coli* χ7122 genome), the *pgl* operon consisting of three fragments each of ~ 5 kb (Pgl1-3), and a gene encoding a kanamycin selection marker surrounded by FRT sited for FLP-mediated marker removal, were each designed to contain a 25 bp overlapping region between each adjoining junction. Each fragment was PCR amplified with Q5 polymerase using oligonucleotide primers listed in Additional file 1: Table S2. The PCR amplified fragments were then assembled into the pCVD422 backbone using the NEB HiFi assembly kit (#2623, New England Biolabs) according to the manufacturer’s instructions. Assembly reactions were transformed into electrocompetent *E. coli* S17-1 λ *pir*. Transformants were selected on LB agar with kanamycin (50 µg/ml). PCR screening, using primers listed in Additional file 1: Table S2, was performed after the transformation to identify positive clones. Plasmids from positive clones were isolated by plasmid miniprep kit (#27104, QIAprep Spin Miniprep Kit, Qiagen). Constructs were confirmed and validated by IIlumina paired end sequencing as described below.

#### Construction of G-NetB/unG-NetB-encoding plasmids

*C. perfringens* NetB (Uniprot ID: A8ULG6) was genetically detoxified (W262A) as described in [61]. The sequence was further modified, with 2 or 10 engineered *N*-glycosylation sites, 5 at the N-terminus and 5 at the C-terminus (or 1 and 1), the native signal peptide was replaced by a PelB signal for secretion to the periplasm, and a C terminal 6xHis tag was added. unG-NetB was designed in the same way, except for the addition of 10 non-functional glycosylation sequons. DNA-encoding the modified NetB versions was commercially synthesised and subcloned into the pEXT20 vector by *Eco*RI-*Pst*I restriction enzyme digest and ligation. G/unG-NetB and the *rpsM* promoter were separately amplified and linked via overlapping PCR. The resulting amplicon was *Eco*RI-*Hind*III digested and subcloned into pFPV25.1.

### pSEC*pgl* sequence verification by Illumina paired-end sequencing

Sequences of putative pSEC*pgl* plasmids were analysed by Illumina paired-end 300 nt long read sequencing. Briefly, plasmid DNA from the PCR and restriction digest-positive colonies was fragmented to 400 bp length using Covaris M220 focused ultrasonicator. Fragmented plasmid DNA were tagged with sequencing adapters using NEBNext UltraII DNA Library Prep Kit for Illumina (#E7645, New England Biolabs). Tagged DNA was then cleaned with AMPure beads, and subsequently indexed with indexing primers with a 12-cycle PCR, cleaned with AMPure beads, then quantified by using NEBNext Library Quant Kit protocol (#E7630, New England Biolabs). Finally, 20 pM quantified libraries were loaded on a MiSeq paired end 2 × 300 nt (MiSeq reagent kitV3 (600 cycles) and sequenced using an Illumina MiSeq run. Raw plasmid sequencing data are available upon request.

Adapter and low quality sequences were trimmed by cutadapt (version 1.16) and sickle tools respectively [83, 84] for all sequencing data. Trimmed reads from each sample were mapped to an in silico generated reference plasmid, pSEC*pgl* sequence using the Burrows Wheeler transform algorithm-mem (version 0.6) read mapper [85] and variants were called using SAMtools (version 1.6) mpileup function [86] with at least 100 × read depth. The output from the variant calling was then used to generate consensus calling and fasta file generation using bcftools consensus function [86] and seqtk seq function (to convert fastQ files to fasta files) [87]. Variant called consensus fasta files of each sample were then annotated using “Feature” function of SnapGene (version 5.2) using an *in-silico* generated pSEC*pgl* plasmid as the reference sequence.

### Integration of *C. jejuni pgl* locus into the genome of APEC χ7122

The bacterial strains used in this study are listed in Table 1. The integration of the *C. jejuni pgl* operon into the genome of APEC χ7122 was achieved by homologous recombination by conjugative delivery of the donor suicide plasmid carrying the *pgl* operon. Briefly, conjugative donor strain, *E. coli* S17-1-λ*pir* carrying pSEC-Pgl5 plasmid and the recipient strain*,* APEC χ7122 were grown to OD_600nm_ of 1.0, mixed at 1:1 ratio in a 200 µl volume and then washed once with 1 × sterile PBS at 3000 rpm for 3 min, resuspended in the 20 µl of 1 × PBS, spotted onto 0.45 µm pore size membrane filter (MF-Millipore 0.45 µm filter, Millipore Sigma) preincubated on a LB agar plate. Spotted membrane filter on LB agar plate was incubated at 37 °C for 4 h to allow conjugation. After 4 h of incubation, the mating filter was resuspended in 1 ml LB broth, serially diluted and plated onto the LB agar containing kanamycin and nalidixic acid at 50 and 25 µg/ml respectively to select co-integrants (only χ7122 recombinants of pSEC*pgl* will grow). Colonies that grew on the LB agar with kanamycin and nalidixic acid were patch plated onto LB agar containing kanamycin and 5% sucrose to select for cells that had undergone a second homologous recombination event which removes the vector sequence and leaves the *pgl* operon integrated in the chromosome. Colonies that were kanamycin and sucrose resistant were tested for loss of vector sequences by patch plating onto LB agar with ampicillin (100 µg/ml). Finally, 10 colonies that were kanamycin and sucrose resistant, and ampicillin sensitive were confirmed by colony PCR for integration of the *pgl* operon terminally marked with kanamycin into the genome between base coordinates 3997780 and 3997791 using primers listed in the Table 2.

### Whole genome sequencing of χ7122 *pgl* integrants

Whole genome sequences of χ7122 wt strain and χ7122 *pgl* integrant were sequenced by Illumina paired end 300 nt long reads. Briefly, genomic DNA from the wild-type strain and four clones of colony PCR positive χ7122 *pgl* integrants were fragmented to 400 bp length using Covaris M220 focused ultrasonicator. Fragmented genomic DNA were tagged with sequencing adapters using NEBNext UltraII DNA Library Prep Kit for Illumina (#E7645, New England Biolabs). Tagged DNA was then cleaned up with AMPure beads, and subsequently indexed with indexing primers with a 12-cycle PCR, cleaned with AMPure beads, then quantified by using NEBNext Library Quant Kit protocol (#E7630, New England Biolabs). Finally, 20 pM quantified libraries were loaded on a MiSeq paired end 2 × 300 nt (MiSeq reagent kitV3 (600 cycles) and sequenced in Illumina MiSeq run. Raw genome sequencing data are available upon request.

Adapter and low-quality sequences were trimmed by cutadapt (version 1.16) and sickle tools respectively for all sequencing data. Trimmed reads from wild-type strain were mapped to published reference genome, NZ_HE962388 [1] and *pgl* integrants were mapped to in silico generated reference, NZ_HE962388_Pgl sequence by Burrows Wheeler transform algorithm-mem (version 0.6) read mapper and PCR duplicates were removed by using PICARD tools. The variants were called using SAMtools (version 1.6) mpileup function with at least 100 × read coverage. Output from variant calling were then used to filter the variants at 100 × read depth using GATK variant filtration function. Finally, filtered variants were annotated by snpEff tool.

### RNA-sequencing

LB broth with appropriate antibiotics was inoculated 1:1000 with an overnight culture of APEC χ7122 wt and χ7122 *pgl*. Three independent cultures were incubated until mid-log phase (OD_600nm_ 0.6–0.8) and harvested. RNA was extracted using the Qiagen miRNeasy kit (following manufacturer’s protocol) with an additional 5-min lysozyme lysis step prior to the addition of Qiazol. rRNA was depleted prior to sequencing library preparation and each library was sequenced to a depth of 10 million 2 × 150 bp paired end reads.

Post sequencing data manipulation and analysis was completed using bowtie2, samtools, FeatureCounts [88], DeSeq2 [89] and R Studio. A reference genome file was generated using the χ7122 HE962388.1 genome file [45] and bowtie2. The RNAseq reads were aligned to this reference file using bowtie2. The aligned read counts for each gene feature were assessed using FeatureCounts, which provides an output file with each gene present in the genome and the number of reads associated with each gene. The DeSeq2 analysis pipeline (R package) was used to assess differential gene expression, with volcano plots being generated in Rstudio.

### Glycoconjugate expression and purification

Overnight pre-cultures grown in LB media with the required antibiotics were diluted 1:100 in fresh 20 ml of LB media in 50 ml closed tubes. Cultures were grown at 37 °C, 180 rpm to OD_600nm_ ~ 0.8 at which point expression of inducible proteins was promoted by the addition of 0.04 (w/v) L-arabinose (for pEC415 and pMAF10 vectors) or by 1 mM IPTG (for pEXT20 vectors). Cultures were then grown at 28 °C for further 16 h on an orbital shaker platform with a rotational speed of 180 rpm, followed by cell harvesting by centrifugation at 5300 g, 4 °C for 15 min.

For His-purifications pellets were resuspended in 1 ml ice-cold lysis buffer (50 mM NaH_2_PO_4_, 300 mM NaCl, and 10 mM imidazole, pH 8.0). Resuspended cells were subjected to six rounds of mechanical lysis using a FastPrep™ homogeniser (30 s per cycle at a speed of 6 m/s). Lysates were clarified by centrifuging at max speed for 10 min on a table-top centrifuge. Clarified lysates were incubated at 4 °C on a roller with 50 µl NiNTA resin (Qiagen). Samples were then transferred to paper filter spin cups (Pierce) and washed 5 × with 500 µl washing buffer (50 mM NaH_2_PO_4_, 300 mM NaCl, 20 mM imidazole pH 8.0). His-tagged proteins/glycoproteins were then purified by competitive elution with 100 µl high imidazole-containing buffer (50 mM NaH_2_PO_4_, 300 mM NaCl, 250 mM imidazole pH 8.0). Glycoconjugate production was verified by SDS-PAGE followed by western blotting and ELISA as described below.

Lysozyme-mediated periplasmic extraction was performed according to [25]. Periplasmic extracts were then used for SDS-PAGE and western blot analysis.

### SDS-PAGE and western blotting

His-pulldowns and OD_600nm_-matched periplasmic extracts were mixed with LDS sample buffer and denatured at 95 °C for 10 min before being separated on 4–12% bis–tris gels in MOPS or MES buffer (Invitrogen, USA). Gels were then electroblotted onto a nitrocellulose membrane, which was then washed in PBS with 0.1% Tween-20 (PBS-T) and incubated overnight at 4 °C shaking in PBS-T with biotin-conjugated SBA lectin (Vector Laboratories) at a 1: 5000 dilution and mouse anti-His monoclonal antibody (Thermo Fisher Scientific) at a 1: 5000 dilution to detect *C. jejuni* heptasaccharide and His-tagged carrier proteins, respectively. Blots were washed 3 × in PBS-T, followed by incubation for 30 min-1 h at room temperature with secondary streptavidin IRDye 800CW and goat anti-mouse IgG IRDye 680 (LI-COR Biosciences) at 1:5000 and 1:10,000 dilution, respectively. After 3 final washes, fluorescent signals in two channels, 700 and 800 nm, were detected with an Odyssey CLx LI-COR detection system (LI-COR Biosciences). Subsequent semi-quantitative densitometry analysis of the glycoconjugates was performed using the Image Studio analysis tool (LI-COR Biosciences). For On-cell western blotting (OCW) cultures were harvested and washed 3 times with PBS, fixed with 4% paraformaldehyde (PFA) in PBS (pH 7.4) for 20 min at room temperature (RT), washed 4 times with PBS, OD_600nm_-matched and spotted in triplicates in a bottom clear 96-well plate. Cell permeabilization of permeabilised samples was performed as described in [90]. Cells were heat fixed and air-dried, washed with PBS-T (0.1% Tween-20) for 2 min static and 2 min shaking at 500 rpm and blocked for 30 min at RT with Carbo-free blocking buffer per well (Vector Laboratories) followed by incubations with primary and secondary antibodies as described above both used at 1:500 dilutions spaced and followed by 3 washes in PBS-T prior to LiCOR acquisition of fluorescent signals.

### Dot-blotting

1 ml of OD_600nm_-matched cultures was pelleted by centrifugation at 12,100 g for 2 min. Pellets were washed 3 × in PBS and resuspended in 100 µl PBS. 2–4 µl were spotted on a nitrocellulose (NC) membrane, let dry and incubated with the respective primary and secondary detection systems as described above.

### Sandwich and whole cell ELISA

In sandwich ELISAs transparent polystyrol 96-well plates with high protein binding capacity (F96 MaxiSorp, Nunc) were coated with mouse anti-his monoclonal antibody (Thermo Fisher Scientific) diluted 1:1000 in PBS (100 µl/well) and incubated overnight at 4 °C. Wells were then washed four times with 200 μl PBS-T (0.1% Tween-20) for 2 min static and 2 min shaking at 500 rpm and blocked for 30 min at RT with 200 μl Carbo-free blocking buffer per well (Vector Laboratories). After blocking, wells were washed twice as described above and eluates from His-pulldowns were diluted 1:100 in PBS. 50 µl/well of diluted eluates were added to the plate and incubated for 1 h at 37 °C. Unbound sample was removed with four washes performed as described above. Semi-quantitative analysis of the glycan content of His-purified *Campylobacter* glycoconjugates were performed using biotin-conjugated SBA lectin (Vector Laboratories) at a 1: 1000 dilution in PBS-T (100 µl/well). The plate was incubated for 30 min at RT, 500 rpm. Unbound lectin was removed by four washes as previously described. Streptavidin-HRP (Invitrogen) was added at 1:10,000 dilution in PBS-T (100 µl/well). The plate was incubated for 30 min at RT, and unbound antibodies were removed by four washes. ELISA plates were developed with 100 µl/well Tetramethylbenzidine (TMB) substrate solution (Invitrogen). The oxidative reaction was stopped by adding 100 µl/well H_2_SO_4_ (2 N) and optical densities at 450 nm were detected using a SpectraMax iD5 plate reader (Molecular Devices). OD_450nm_ background values (buffer only in wells treated with bioSBA lectin and streptavidin-HRP) were subtracted from test values. Technical and biological triplicates were averaged, values represent the arithmetic mean and error bars represent standard deviations of biological triplicates. For whole cell ELISAs, cultures were harvested, washed and resuspended in PBS to OD_600nm_ ~ 0.4. Cell resuspensions were spotted in triplicates in transparent polystyrol 96-well plates (F96 MaxiSorp, Nunc) at 100 µl/well and incubated overnight at 4 °C. Blocking, washes, primary and secondary antibodies staining and development were performed as described above. Surface display of NetB was detected with anti-His at 1:2000 dilution followed by anti-mouse IgG-HRP at 1:10,000. Surface display of *C. jejuni* heptasaccharide was detected with SBA lectin as described above.

### Animal experiments

White Leghorn chickens from *Campylobacter*-free flocks were obtained on the day of hatch from a Home Office licensed breeding establishment and housed in groups of up to twenty in colony cages. Groups were of mixed sex and individuals were wing-tagged for identification. Water and sterile irradiated feed based on vegetable protein (DBM Ltd., UK) were provided ad libitum. Animal experiments were conducted at the Moredun Research Institute according to the requirements of the Animals (Scientific Procedures) Act 1986 under project licence PCD70CB48 with the approval of the local Animal Welfare & Ethical Review Board. Chickens were monitored twice daily, or at least every 3 h when birds presented signs of colibacillosis. Post-mortem examinations were conducted following culling by cervical dislocation.

#### Determining the in vivo fitness of the APEC pgl integrant

Fitness of individual strains was determined in an intra-air sac challenge model in which groups of ten chickens were directly inoculated in the air sac with 100 μl of culture containing 3 × 10^6^ CFU of the χ7122 wt or χ7122 *pgl* strain. Post-mortem examinations were performed at 8 h post-challenge on the appearance of clinical signs of colibacillosis. The lungs were collected to determine net replication close to the site of inoculation and the liver and spleen were collected to quantify systemic translocation. 0.5 g of tissue was homogenised in 4.5 ml PBS. Serial dilutions of the homogenates were plated on LB agar with strain-appropriate antibiotics and incubated overnight at 37 °C to determine the viable counts. Fitness of strains was also tested in an oral challenge model in a competition assay in which groups of 10 chickens were orally gavaged with 100 μl of a mixed culture containing 1.5 × 10^5^ and 1.5 × 10^7^ CFU of both χ7122 and χ7122 *pgl*. Post-mortem examinations were performed for five chickens from each group at 2 and 7 days post-challenge. The caecal contents and livers were collected, serially diluted as above and plated on both LB agar with only nalidixic acid to enumerate both χ7122 and χ7122 *pgl* and LB agar with nalidixic acid and kanamycin to enumerate only χ7122 *pgl*. Plates were incubated overnight at 37 °C to determine the viable counts from which the competitive indices were calculated as the ratio of mutant to wild-type in output pools divided by the ratio of mutant to wild-type in the challenge inoculum.

#### Vaccination with χ7122 pgl pEXT20-G-NetB(10)

χ7122 *pgl* pEXT20-G-NetB(10) was grown overnight at 37 °C in LB broth Lennox, diluted 1:100 in fresh broth with the required antibiotics and grown until an OD_600nm_ ~ 0.8 was reached. At this point G-NetB(10) expression from pEXT20 was induced with 1 mM IPTG and the cultures were incubated at 28 °C shaking (180 rpm) overnight. The next day cultures were diluted to approximately 10^8^ CFU/ml and glycoprotein production in this challenge inoculum was confirmed by western blotting. To evaluate the efficacy of χ7122 *pgl* pEXT20-G-NetB(10) as a vaccine against caecal colonisation by *C. jejuni* strain M1, chickens were orally vaccinated with 100 μl of this culture containing 10^7^ CFU at days 7 and 21 of life. Mock vaccinated chickens were orally gavaged with 100 μl LB broth. At day 21, before the second vaccination, and at day 28, before challenge with *C. jejuni* M1, post-mortem examinations were performed for three or four chickens per group to determine colonisation levels of χ7122 *pgl* pEXT20-G-NetB(10). The caecal contents were collected, serially diluted and plated on LB agar with only kanamycin to detect the vaccine strain and kanamycin and ampicillin to confirm maintenance of pEXT20-G-NetB(10). Plates were incubated overnight at 37 °C to determine the viable counts. Liquid cultures of *C. jejuni* M1 were prepared as above and diluted to obtain a challenge dose of 100 CFU. Chickens were orally gavaged with this inoculum at day 28 and post-mortem examinations were performed on day 35. The caecal contents were collected, serially diluted and plated on CCDA agar to determine colonisation levels of *C. jejuni* M1.

#### Vaccination with χ7122 pgl pFPV25.1-G-NetB(10)/unG-NetB

Ninety chickens were divided into three vaccination groups according to the vaccine strain administered: χ7122 *pgl* pFPV25.1-G-NetB(10), χ7122 *pgl* pFPV25.1-unG-NetB and mock-vaccinated group. Each group was housed in a different room to prevent cross contamination between vaccination strains and further divided into four subgroups of: (a) 10 birds vaccinated orally and challenged with *C. jejuni* 11168H, (b) 10 birds vaccinated by the intra-air sac route and challenged with *C. jejuni* 11168H, (c) 5 birds vaccinated orally and challenged with wild type χ7122, and (d) 5 birds vaccinated by intra-air sac and challenged with wt χ7122. Orally vaccinated chickens were inoculated with 100 μl of χ7122 *pgl* pFPV25.1-G-NetB(10) or χ7122 *pgl* pFPV25.1-unGNetB containing 10^7^ CFU at days 7 and 21 of life. Mock vaccinated chickens were orally gavaged with 100 μl of PBS. At the same time points, two additional groups were vaccinated by the intra-air sac route with 100 μl of the vaccine strains containing 10^3^ CFU of the APEC strains and mock-vaccinated chickens were treated with 100 μl of PBS. At day 28, ten chickens that were vaccinated orally or by the intra-air sac route were challenged by oral gavage with 4 × 10^5^ CFU of *C. jejuni* 11168H. Post-mortem examinations of these chickens were performed at day 35 as described above and the caecal contents were collected, serially diluted and plated on CCDA agar to determine colonisation levels of *C. jejuni* 11168H. At day 36, the remaining five chickens in each group were challenged by the intra-air sac route with 100 μl of *E. coli* χ7122 wt containing 7 × 10^6^ CFU. Post-mortem examinations of these chickens were performed at day 37 as described above. The lungs were collected, serially diluted and plated on MacConkey agar to determine colonisation levels of *E. coli* χ7122.

### Serological ELISA

Blood was collected by cardiac puncture at post-mortem (PM) examination and serum was stored at − 80 °C following centrifugation of clotted blood at 1000 g for 10 min at 4 °C. Serum IgY levels were quantified by ELISA as described. To quantify vaccine-specific responses, 96-well plates were coated with 0.1 μg/ml of purified unG-NetB or G-NetB(10) antigens in carbonate-bicarbonate buffer and incubated at 4 °C overnight. Plates were washed three times with PBS containing 0.05% (v/v) Tween 20 and 50 μl of 1:50 serum diluted in PBS was added per well. Control wells were maintained to which no serum was added. Plates were incubated at room temperature for 1 h and then washed as above. Rabbit anti-chicken IgY-horseradish peroxidase (HRP) antibody at 1:3000 (Sigma, UK) was used to detect bound serum IgY. Plates were washed three times, tetramethylbenzidine (TMB) substrate (BioLegend, UK) was added and the plates were incubated for 10 min at room temperature in the dark. The reaction was stopped using 2 M H_2_SO_4_ and optical density at 450 nm adjusted against optical density at 620 nm (OD_450/620 nm_) was measured using a plate reader (Biotek Cytation 3) with background correction using the values of the no serum control wells.

### Statistical analysis

Statistical analyses of competitive indices and differences in colonisation between groups of pEXT20-G-NetB(10) vaccinated and mock-vaccinated chickens were performed in GraphPad Prism version 8.00 (GraphPad Software) using the Mann–Whitney U test. P values < 0.05 were considered to be statistically significant. Data are represented graphically as median values with 95% confidence intervals. Bacterial counts for unbalanced data from the animal trial using pFVP25.1-based vaccines were analysed in Minitab version 19 (Minitab, LLC), using a general linear model (GLM) and post-hoc Dunnett method. Two tailed t-tests for analysis of densitometry and ELISA data were performed using Microsoft Excel (version 2013).

## Supplementary Information

**Additional file 1**: **Figure S1**. Exploiting G-ExoA constructs to test N-linked glycosylation in χ7122. (a) Schematic representation of carrier protein ExoA genetically detoxified and modified with either two, G-ExoA(2), or ten ,G-ExoA(10), PglB glycosylation sequons. (b) SDS-PAGE followed by western blotting showing results of a His-pulldown from χ7122 expressing G-ExoA(2) or G-ExoA(10) and the pgl locus from pACYCpgl plasmid. Glycosylation increases as the number of glycosylation sequons increases, facilitating detection of glycosylation with G-ExoA(10). The negative control (- ctrl) consists of χ7122 expressing G-ExoA(10) in absence of the pgl locus. Percentage in parenthesis indicates sample loading (v/v) per lane. **Figure S2**. Identification of conditions that favour protein glycosylation in the χ7122 pgl integrant. (a) SDS-PAGE followed by western blotting showing results of a His-pulldown from the χ7122pgl integrant transformed with a plasmid-encoded L-arabinose inducible G-ExoA(10) carrier protein. Differences in culture conditions are enlisted in the table below the blot. (b) SDS-PAGE followed by western blotting showing results of a His-pulldown from either glycoengineering strains SDB1 or χ7122 pgl integrant transformed with a plasmid-encoded L-arabinose inducible G-ExoA(10) carrier protein and with pACYCpgl for SDB1. Both strains were cultured in either LBB Lennox or 2YT-M9 media to assess their effect on glycosylation. The most favourable conditions for protein glycosylation in χ7122 pgl were identified as cultures setup in a closed system (20 ml in 50 ml tube), induction of carrier protein expression at late exponential phase (OD600 ~0.8), growth post induction at 28°C shaking in LBB Lennox medium. Percentages in parenthesis indicate sample loading (v/v) per lane. **Figure S3**. Testing functionality of χ7122 pgl integrants. SDS-PAGE followed by western blotting of periplasmic extracts shown in Figure 3b. Fluorescent signals were acquired from single channels at 680 nm wavelength for the protein (anti-His) and 800 nm for the glycan (SBA lectin). The boxes highlighted were used for semi-quantitative densitometry analysis of protein and glycan levels. Percentage in parenthesis indicates sample loading (v/v) per lane. **Figure S4**. Assessing G-NetB as a carrier protein. (a) Schematic representation of carrier protein NetB genetically detoxified and modified with either two, G-NetB(2), or ten, G-NetB(10), PglB glycosylation sequons. (b) SDS-PAGE followed by western blotting showing glycosylation of G-NetB(2) or G-NetB(10) by a χ7122 pgl integrant and the glycoengineering strains used as controls. Lanes 1-2 SDB1 cells expressing G-NetB(2), lane 3 CLM24 cedA::pglB cells expressing G-NetB(10), lanes 4-5 SDB1 cells expressing G-NetB(10), lanes 6-7 χ7122 pgl expressing G-NetB(2) and G-NetB(10). pACYCpglΔpglB (in lanes 2 and 5) serves as a negative control on protein glycosylation. In strain CLM24 cedA::pglB (lane 4) pACYCpglΔpglB provides pgl genes responsible for the glycan assembly, while an IPTG-inducible copy of PglB is chromosomally integrated. **Figure S5**. Expression of inducible and constitutive G-NetB/unG-NetB from biological triplicates of χ7122 pgl vaccine strains. (a) SDS-PAGE followed by western blotting showing results of a His-pulldown of biological triplicates of χ7122 pgl vaccine strain expressing G-NetB. Lanes 1-3 G-NetB expressed from IPTG-inducible pEXT20 backbone, lanes 4-6 from constitutive pFPV25.1 backbone; (b) SDS-PAGE followed by western blotting showing results of a His-pulldown of biological triplicates of χ7122 pgl vaccine strain expressing unG-NetB as a negative control on glycosylation. Lanes 1-3 unG-NetB expressed from IPTG-inducible pEXT20 backbone, lanes 4-6 from constitutive pFPV25.1 backbone. Percentages in parenthesis indicate samples loading (v/v) per lane. **Figure S6**. PCR validation of the vaccine strains. Agarose gel showing PCR amplicons validating the presence of four endogenous plasmids of strain χ7122 (lanes 1-4) and carrier protein-encoding plasmid pFPV25.1-G-NetB(10) in the G-NetB(10) vaccine strain (lane 5, left), and unG-NetB in the unglycosylatable control (lane 5, right). **Figure S7**. Purified G-NetB(10) and unG-NetB for use as coating antigens in serological ELISAs. Comassie (a) and SDS-PAGE followed by western blotting (b) of purified fractions of G-NetB(10) and unG-NetB. The glycoprotein and its unglycosylated control where purified by His-affinity chromatography and anion exchange on an AKTÄ purifier, followed by size exclusion chromatography using PD-10 gravity columns. Fraction 1 of G-NetB(10), which is entirely glycosylated at 1 to 10 sites and has no degradation product was used in ELISA together with unG-NetB to quantify antibody responses against the glycoprotein and protein-only, respectively. 1 µg/lane was loaded. **Table S1**. RNA-Seq: number of reads assigned to each gene within the pgl locus. **Table S2**. Oligos used in this study

**Additional file 2**: WGS of χ7122 and χ7122 pgl summary

## Data Availability

Genome sequence data and raw RNA-Seq data for the χ7122 *pgl* integrant are available on request and are in the process of submission to the European Nucleotide Archive (https://www.ebi.ac.uk/ena). Accession numbers will be added to the final version of the manuscript. Primary data and images from experiments are available for scrutiny on request. They will be held on secure servers for at least 10 years in accordance with funder requirements. Materials will be made available at no cost but may be subject to a Material Transfer Agreement owing to associated Intellectual Property. We may seek a contribution toward the cost of biosecure shipment of live materials.

## Abbreviations

APEC: Avian pathogenic *E. coli*
bp: Base pairs
CCDA: Charcoal-cephoperazone-deoxycholate agar
CFU: Colony forming units
Da: Dalton
diNAcBac: *N,N′*-Diacetylbacillosamine
ECA: Enterobacterial common antigen
G- (prefix): Glycosylatable
GlcNAc: *N*-Acetylglucosamine
Glc: Glucose
GLM: General linear model
kDa: Kilodalton
kbp: Kilobase pairs
LAV: Live attenuated vaccines
OCW: On-cell western blot
OD: Optical density
ON: Overnight
OST: Oligosaccharyltransferase
PFA: Paraformaldehyde
PGCT: Protein glycan coupling technology
PM: Post-mortem
RT: Room temperature
SBA: Soybean agglutinin
SNP: Single nucleotide polymorphism
Spp.: Species
TMB: Tetramethylbenzidine
unG- (prefix): Unglycosylatable
WCL: Whole cell lysates
WGS: Whole genome sequencing
