# Supplementary material for: Multivalent poultry vaccine development using Protein Glycan Coupling Technology
Source: Microb Cell Fact. 2021 Oct 2;20:193. doi: 10.1186/s12934-021-01682-4 (PMC8487346; doi:10.1186/s12934-021-01682-4)
Supplement: Supplementary file 1 — Additional file 1: Figure S1. Exploiting G-ExoA constructs to test N-linked glycosylation in χ7122. (a) Schematic representation of carrier protein ExoA genetically detoxified and modified with either two, G-ExoA(2), or ten ,G-ExoA(10), PglB glycosylation sequons. (b) SDS-PAGE followed by western blotting showing results of a His-pulldown from χ7122 expressing G-ExoA(2) or G-ExoA(10) and the pgl locus from pACYCpgl plasmid. Glycosylation increases as the number of glycosylation sequons increases, facilitating detection of glycosylation with G-ExoA(10). The negative control (- ctrl) consists of χ7122 expressing G-ExoA(10) in absence of the pgl locus. Percentage in parenthesis indicates sample loading (v/v) per lane. Figure S2. Identification of conditions that favour protein glycosylation in the χ7122 pgl integrant. (a) SDS-PAGE followed by western blotting showing results of a His-pulldown from the χ7122pgl integrant transformed with a plasmid-encoded L-arabinose inducible G-ExoA(10) carrier protein. Differences in culture conditions are enlisted in the table below the blot. (b) SDS-PAGE followed by western blotting showing results of a His-pulldown from either glycoengineering strains SDB1 or χ7122 pgl integrant transformed with a plasmid-encoded L-arabinose inducible G-ExoA(10) carrier protein and with pACYCpgl for SDB1. Both strains were cultured in either LBB Lennox or 2YT-M9 media to assess their effect on glycosylation. The most favourable conditions for protein glycosylation in χ7122 pgl were identified as cultures setup in a closed system (20 ml in 50 ml tube), induction of carrier protein expression at late exponential phase (OD600 ~0.8), growth post induction at 28°C shaking in LBB Lennox medium. Percentages in parenthesis indicate sample loading (v/v) per lane. Figure S3. Testing functionality of χ7122 pgl integrants. SDS-PAGE followed by western blotting of periplasmic extracts shown in Figure 3b. Fluorescent signals were acquired from single channels at [file 12934_2021_1682_MOESM1_ESM.doc]

Appendix 1

# Supplementary Figures, Tables and Methods

## Supplementary Figures


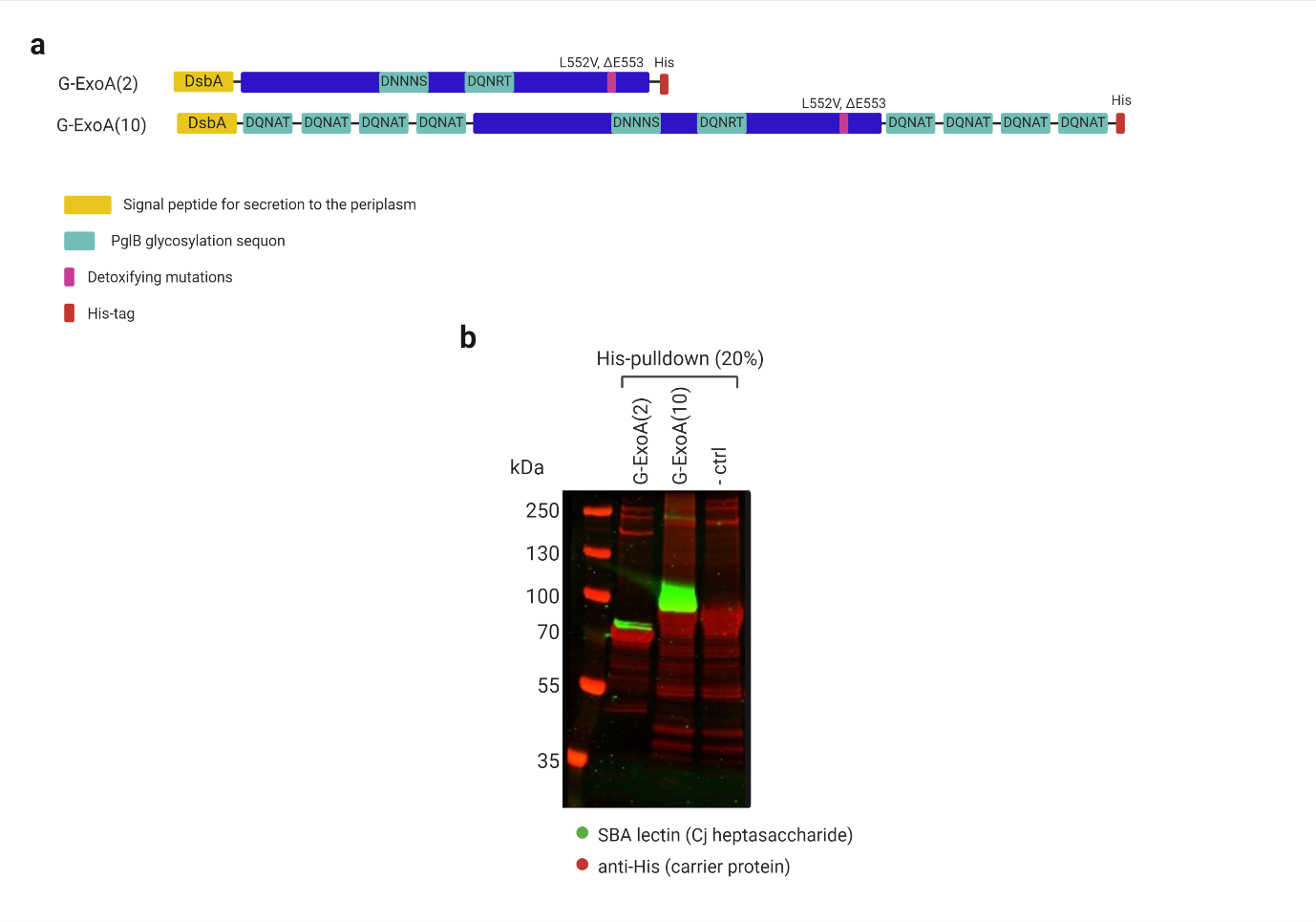


**Figure S1. Exploiting G-ExoA constructs to test N-linked glycosylation in χ7122**

1. Schematic representation of carrier protein ExoA genetically detoxified and modified with either two, G-ExoA(2), or ten ,G-ExoA(10), PglB glycosylation sequons.
2. SDS-PAGE followed by western blotting showing results of a His-pulldown from χ7122 expressing G-ExoA(2) or G-ExoA(10) and the *pgl* locus from pACYC*pgl* plasmid. Glycosylation increases as the number of glycosylation sequons increases, facilitating detection of glycosylation with G-ExoA(10). The negative control (- ctrl) consists of χ7122 expressing G-ExoA(10) in absence of the *pgl* locus. Percentage in parenthesis indicates sample loading (v/v) per lane.


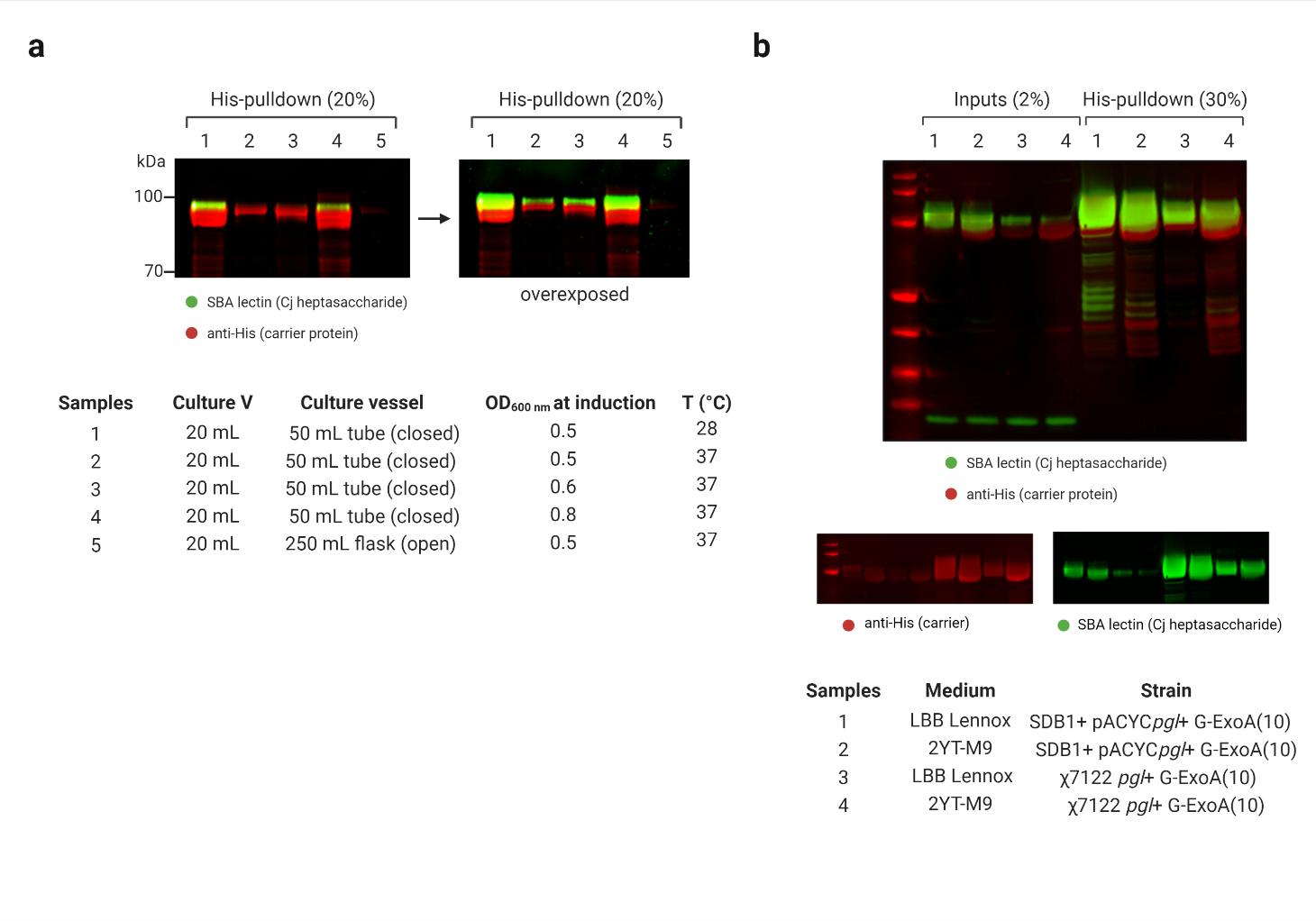


**Figure S2. Identification of conditions that favour protein glycosylation in the** χ**7122 *pgl* integrant**

1. SDS-PAGE followed by western blotting showing results of a His-pulldown from the χ7122*pgl* integrant transformed with a plasmid-encoded L-arabinose inducible G-ExoA(10) carrier protein. Differences in culture conditions are enlisted in the table below the blot.
2. SDS-PAGE followed by western blotting showing results of a His-pulldown from either glycoengineering strains SDB1 or χ7122 *pgl* integrant transformed with a plasmid-encoded L-arabinose inducible G-ExoA(10) carrier protein and with pACYC*pgl* for SDB1. Both strains were cultured in either LBB Lennox or 2YT-M9 media to assess their effect on glycosylation.

The most favourable conditions for protein glycosylation in χ7122 *pgl* were identified as cultures setup in a closed system (20 ml in 50 ml tube), induction of carrier protein expression at late exponential phase (OD_600_ ~0.8), growth post induction at 28°C shaking in LBB Lennox medium. Percentages in parenthesis indicate sample loading (v/v) per lane.


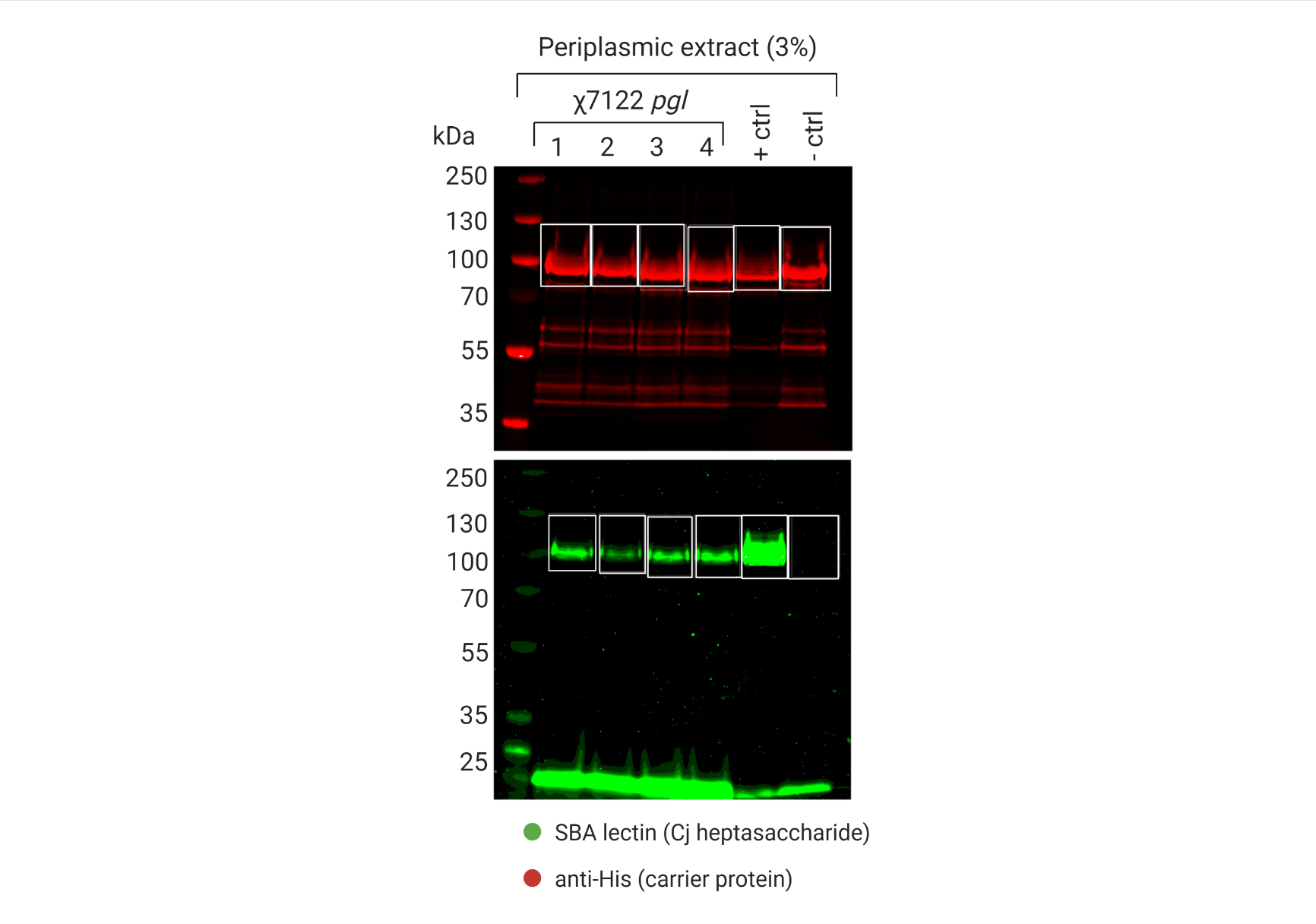


**Figure S3. Testing functionality of χ7122 *pgl* integrants**

SDS-PAGE followed by western blotting of periplasmic extracts shown in Figure 3b. Fluorescent signals were acquired from single channels at 680 nm wavelength for the protein (anti-His) and 800 nm for the glycan (SBA lectin). The boxes highlighted were used for semi-quantitative densitometry analysis of protein and glycan levels. Percentage in parenthesis indicates sample loading (v/v) per lane.


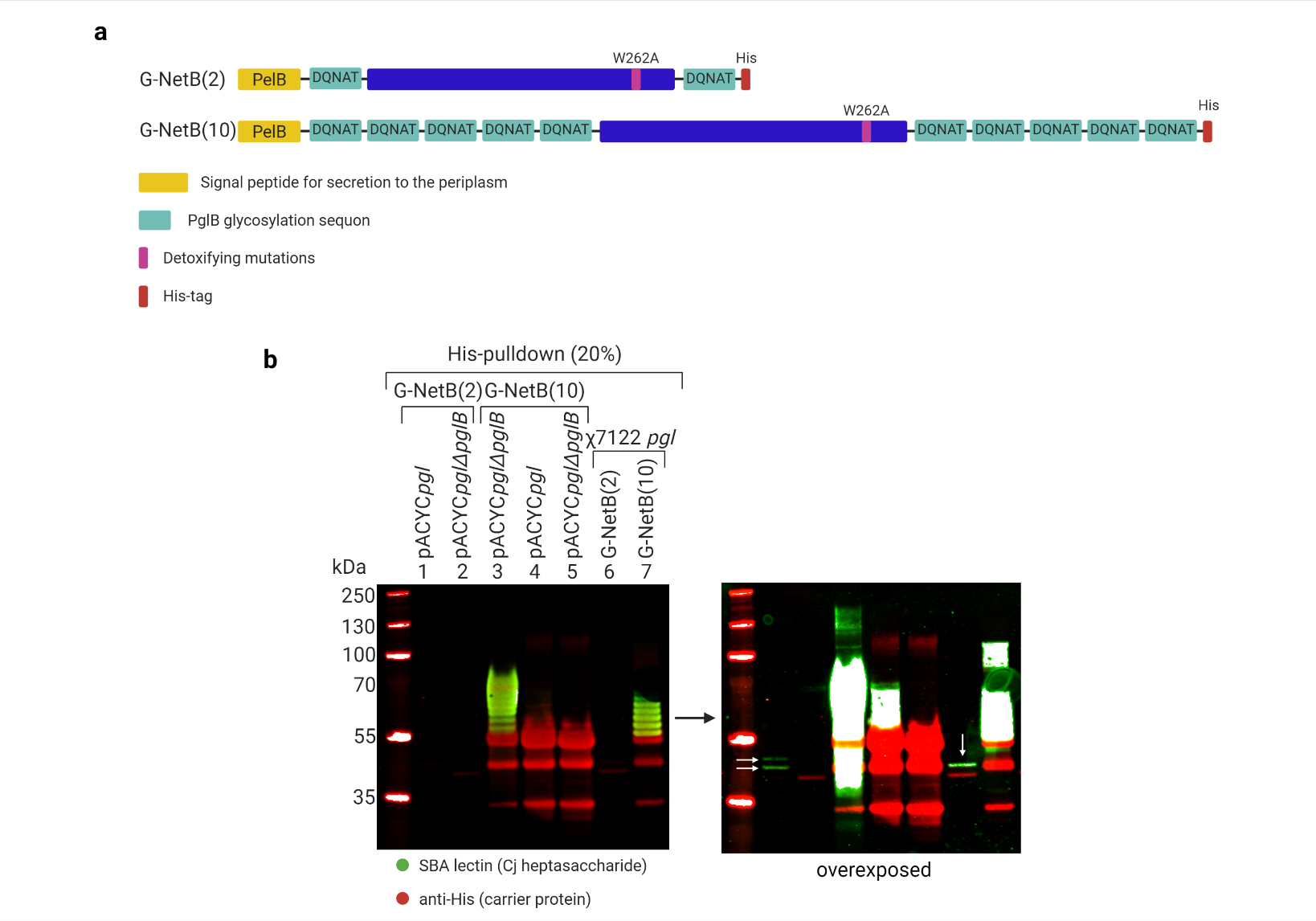


**Figure S4. Assessing G-NetB as a carrier protein**

1. Schematic representation of carrier protein NetB genetically detoxified and modified with either two, G-NetB(2), or ten, G-NetB(10), PglB glycosylation sequons.
2. SDS-PAGE followed by western blotting showing glycosylation of G-NetB(2) or G-NetB(10) by a χ7122 *pgl* integrant and the glycoengineering strains used as controls. Lanes 1-2 SDB1 cells expressing G-NetB(2), lane 3 CLM24 *cedA::pglB* cells expressing G-NetB(10), lanes 4-5 SDB1 cells expressing G-NetB(10), lanes 6-7 χ7122 *pgl* expressing G-NetB(2) and G-NetB(10). pACYC*pgl*Δ*pglB* (in lanes 2 and 5) serves as a negative control on protein glycosylation. In strain CLM24 *cedA::pglB* (lane 4) pACYC*pgl*Δ*pglB* provides *pgl* genes responsible for the glycan assembly, while an IPTG-inducible copy of PglB is chromosomally integrated.


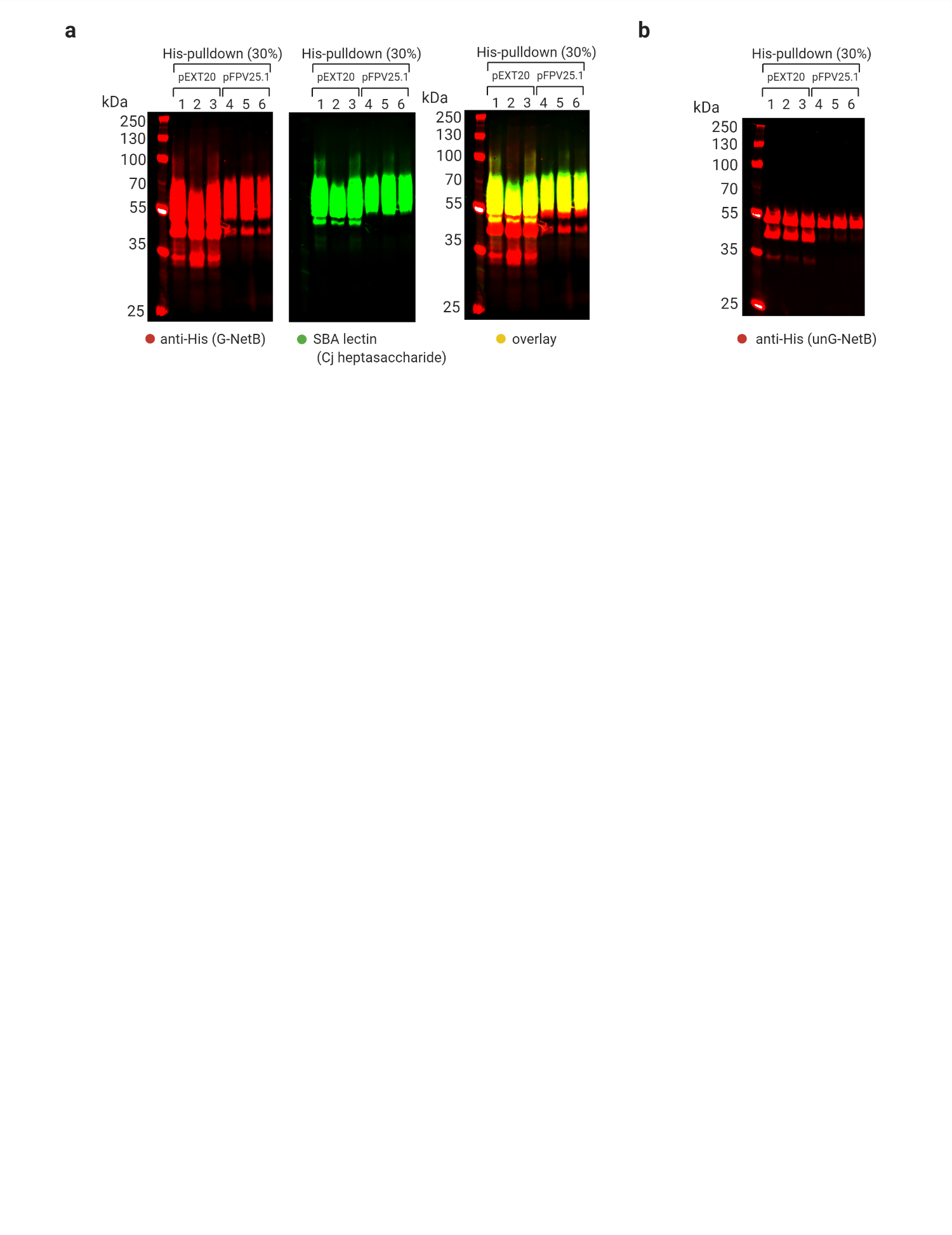


**Figure S5. Expression of inducible and constitutive G-NetB/unG-NetB from biological triplicates of χ7122 *pgl* vaccine strains**

1. SDS-PAGE followed by western blotting showing results of a His-pulldown of biological triplicates of χ7122 *pgl* vaccine strain expressing G-NetB. Lanes 1-3 G-NetB expressed from IPTG-inducible pEXT20 backbone, lanes 4-6 from constitutive pFPV25.1 backbone;
2. SDS-PAGE followed by western blotting showing results of a His-pulldown of biological triplicates of χ7122 *pgl* vaccine strain expressing unG-NetB as a negative control on glycosylation. Lanes 1-3 unG-NetB expressed from IPTG-inducible pEXT20 backbone, lanes 4-6 from constitutive pFPV25.1 backbone.

Percentages in parenthesis indicate samples loading (v/v) per lane.


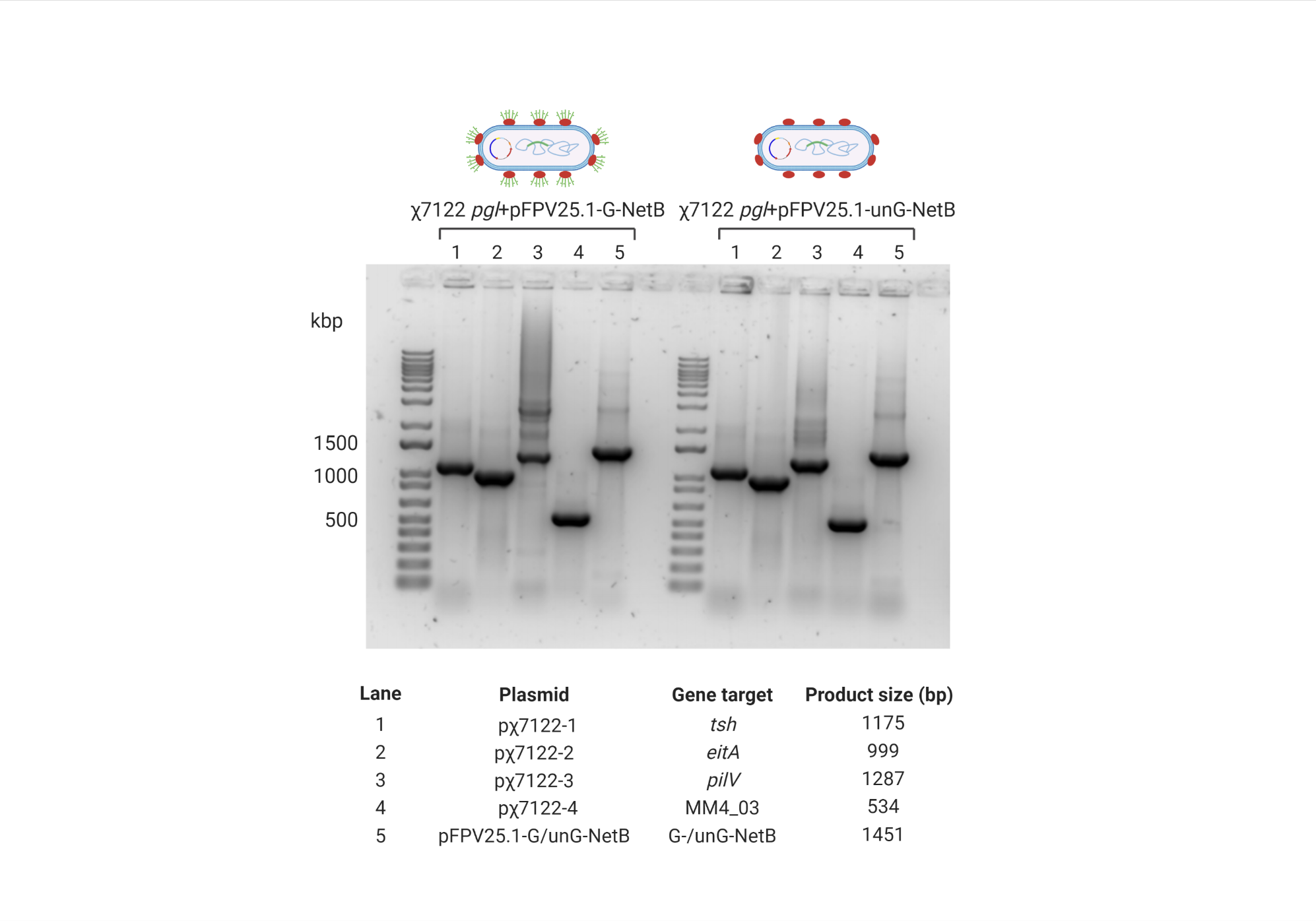


**Figure S6. PCR validation of the vaccine strains**

Agarose gel showing PCR amplicons validating the presence of four endogenous plasmids of strain χ7122 (lanes 1-4) and carrier protein-encoding plasmid pFPV25.1-G-NetB(10) in the G-NetB(10) vaccine strain (lane 5, left), and unG-NetB in the unglycosylatable control (lane 5, right).


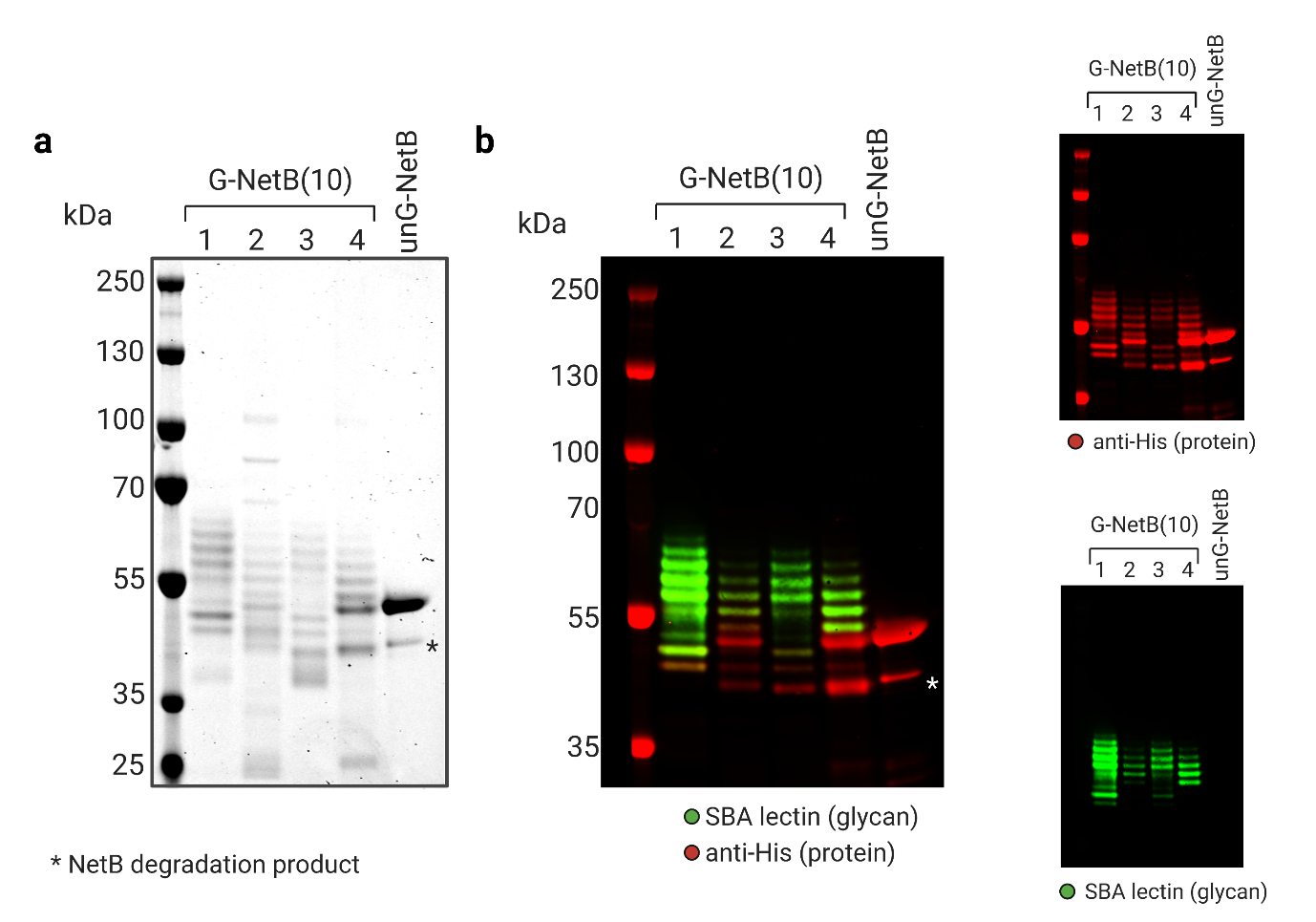


**Figure S7. Purified G-NetB(10) and unG-NetB for use as coating antigens in serological ELISAs**

Comassie **(a)** and SDS-PAGE followed by western blotting **(b)** of purified fractions of G-NetB(10) and unG-NetB. The glycoprotein and its unglycosylated control where purified by His-affinity chromatography and anion exchange on an AKTÄ purifier, followed by size exclusion chromatography using PD-10 gravity columns. Fraction 1 of G-NetB(10), which is entirely glycosylated at 1 to 10 sites and has no degradation product was used in ELISA together with unG-NetB to quantify antibody responses against the glycoprotein and protein-only, respectively. 1 µg/lane was loaded.

## Supplementary Tables

**Table S1. RNA-Seq: number of reads assigned to each gene within the *pgl* locus**

|  | **χ7122 *pgl*** | | |
| --- | --- | --- | --- |
| **Genes** | **Replicate 1** | **Replicate 2** | **Replicate 3** |
| ***pglG*** | 663 | 551 | 1015 |
| ***pglF*** | 1243 | 1166 | 2001 |
| ***pglE*** | 1087 | 970 | 1761 |
| ***pglD*** | 497 | 506 | 802 |
| ***pglC*** | 374 | 339 | 516 |
| ***pglA*** | 1579 | 1403 | 2149 |
| ***pglB*** | 3373 | 2895 | 4993 |
| ***pglJ*** | 1957 | 1743 | 2793 |
| ***pglI*** | 2144 | 1982 | 2826 |
| ***pglH*** | 854 | 839 | 1301 |
| ***pglK*** | 397 | 371 | 547 |
| ***galE*** | 1975 | 1741 | 2198 |
| ***wlaX*** | 740 | 683 | 860 |
| ***waaC*** | 193 | 184 | 242 |
| **Sum** | **17076** | **15373** | **24004** |
| **Total # reads per library** | **47410696** | **50018467** | **48144224** |

**Table S2. Oligos used in this study**

| **Oligo** | **5’ to 3’ Sequence** |
| --- | --- |
| TS246 | CCATTTGCCTGCTTTTATA |
| TS247 | ATATCAATGATTTTCTGGTG |
| TS458 | TCTCAACCCGGTACGCACCAGAAAATCATTGATATGACGTCCGCATAACGCATTAAAAAT |
| TS459 | ATAATGCAGTAATAGATCACTAGTTCAGTCTAAAAACAGATACT |
| TS460 | TATCTGTTTTTAGACTGAACTAGTGATCTATTACTGCATTATAGTTGTTTTTTC |
| TS431 | CACCACACTTTTAGTATCATCACTAGAATTATCATCGCTTACA |
| TS432 | GCGATGATAATTCTAGTGATGATACTAAAAGTGTGGTG |
| TS433 | TTAGCATTTTTTGTTTTTGCCCAAAGTCCATCATAAG |
| TS434 | TTATGATGGACTTTGGGCAAAAACAAAAAATGCTAAGGA |
| TS435 | TCAAGACGTGTAATGCGTTATGGCAATGGTACTTTCA |
| TS436 | AAGTACCATTGCCATAACGCATTACACGTCTTGAGC |
| TS461 | GCATATTTATTTACTTGGGCGGCCGCTTAGCCATGGTCCATATG |
| TS462 | CATATGGACCATGGCTAAGCGGCCGCCCAAGTAAATAAATATGCTGTG |
| TS463 | AATCCCATATGAACTATATAAAAGCAGGCAAATGGCTCGAGGGTACATGTCTTTCACGC |
| TS442 | GAAGCATTTATCAGGGTTAT |
| TS443 | TATAGAAATTCATTGGTTTGTAG |
| TS444 | GCTTTGCCTTTGTTCTTTT |
| TS445 | CTATGAGCATTTTTACTCGC |
| TS446 | GAGTTTATGCGGAATTTAGG |
| TS447 | ATTCATCGCTTAATAACTCA |
| TS448 | TCGTTCTTATTTTTATAGAGC |
| TS449 | CAATCCATCTTGTTCAATC |
| TS450 | TTCTATGAAAGGTTGGGCT |
| TS451 | TTCTACGCAGACAAACAAT |
| EcoRI-PelB-G-/unG-NetB fw | aaatagaattcATGAAATATCTGCTGCCGAC |
| PstI-stop-6xHis-G-/unG-NetB rev | aaatactgcagcTTAATGGTGATGATGATGATG |
| EcoRI-rpsM-F | CAAGAATTCGAGCTCGGTACCCGGGGAT |
| rbs-rpsM-R | ATGTATATCTCCTTCTTAAATCTAGAGGATCTTAACATTTTCAGCGATA |
| rbs-PelB-F | ATTTAAGAAGGAGATATACATATGAAATATCTGCTGCCGACCGCAG |
| HindIII-NetB-6H-R2 | CAGAAGCTTGCATGCCTGCAGCTTAATGGTGATGATGATGA |
| 7122-1-tsh-F | ATGAACAGAATTTATTCTCTTC |
| 7122-1-tsh-R2 | TTAGAGGTTGTTACTGTGTAAT |
| 7122-2-eitA-F | ATGAAGAAGGTCCTTTGCGCGTT |
| 7122-2-eitA-R | TCAGTTCAGCCCCATTTTTCTCAGC |
| 7122-3-pilV-F | ATGAAAAAGACAGATAAAGGTGTATCTCTCCTTGA |
| 7122-3-pilV-R | TCACTGGCAAATGGCGTAAACTGT |
| 7122-4-MM4_03-F | ATGATTTTACGGTCTCACACGCAG |
| 7122-4-MM4_03-R | TTACGCACTGTCTTTGGTTAGTAGCT |
| FPV25.1-F1 | GCGTTAACTTCGATCTACGGTGT |
| FPV25.1-R1 | CGATATCCCGCAAGAGGCCCGG |

## Supplementary Methods

### Purification of G-NetB(10) and unG-NetB as coating antigens for serological ELISA

Plasmids pEXT20-unG-/G-NetB(10) were introduced by electroporation into a modified *E. coli* W3110-derivative carrying a chromosomal copy of the *pgl* locus. Transformants were grown overnight at 37°C in lysogeny broth (LB) with the appropriate antibiotics. The following day, cultures were diluted 1:100 and 200 ml starters were grown at 37 °C under shaking conditions (180 rpm). Next day starters were inoculated in a 2 l culture. At OD_600 nm_~ 0.8, 1 mM IPTG was added to induce NetB expression and cultures were grown for other 16 h at 28 °C. Cells were harvested by centrifugation at 5400 *g* for 30 minutes at 4 °C, resuspended in ice-cold lysis buffer (50 mM NaH_2_PO_4_, 300 mM NaCl, 20 mM imidazole, pH 8.0), homogenized by sonication and mechanically lysed using a pre-chilled Stansted High Pressure Cell Disruptor (Stansted Fluid Power Ltd.) under 60,000 psi (410 MPa) in continuous mode for five rounds. The lysate was treated with 25 U/ml Benzonase (Sigma) and clarified by centrifugation at 10,000 g for 1 h at 4 °C. The supernatant was filtered (0.22 µm) and loaded onto the superloop of an AKTÄ purifier (Amersham) for Ni-affinity chromatography using a HisTrap HP column (Cytiva). The column was then washed 10x with lysis buffer containing 0.1% Triton X-114 and 20x without detergent. The proteins were eluted on a linear gradient in lysis buffer with up to 300mM Imidazole. Protein fractions of interest were pooled, desalted and buffer exchanged using PD-10 columns (GE Healthcare) into 20mM Tris-HCl pH 8.0 for anion exchange (AEx) chromatography using HiTrap Q columns (Cytiva). AEx columns were washed 10x and samples were eluted on a linear gradient up to 1M NaCl in 20mM Tris-HCl pH 8.0. Fractions of interest were pooled, desalted and buffer exchanged using PD-10 columns (GE Healthcare) into PBS. Purity of G-NetB(10) and unG-NetB was assessed by Coomassie staining of proteins resolved by SDS-PAGE, and western blotting. Protein concentration was determined by absorbance at 280 nm measured with NanoDrop (ThermoFisher), using the following parameters: extinction coefficient (ε)= 51,340 M-1 cm-1, Molecular weight (MW) = 40,198.77 Da, A_280 nm_ (0.1%)= 1.28 for G-NetB(10) without PelB, and ε= 51,340 M-1 cm-1, MW = 40,339.03 Da, A_280 nm_ (0.1%)= 1.27 for unG-NetB without PelB.
